# Cladribine and ocrelizumab induce differential miRNA profiles in peripheral blood mononucleated cells from relapsing–remitting multiple sclerosis patients

**DOI:** 10.3389/fimmu.2023.1234869

**Published:** 2023-12-13

**Authors:** Ivan Arisi, Leonardo Malimpensa, Valeria Manzini, Rossella Brandi, Tommaso Gosetti di Sturmeck, Chiara D’Amelio, Sebastiano Crisafulli, Gina Ferrazzano, Daniele Belvisi, Francesca Malerba, Rita Florio, Esterina Pascale, Hermona Soreq, Marco Salvetti, Antonino Cattaneo, Mara D’Onofrio, Antonella Conte

**Affiliations:** ^1^ European Brain Research Institute (EBRI) Rita Levi-Montalcini, Rome, Italy; ^2^ Institute of Translational Pharmacology, National Research Council, Rome, Italy; ^3^ Istituto di Ricovero e Cura a Carattere Scientifico (IRCCS) Istituto Neurologico Mediterraneo Neuromed, Pozzilli, Italy; ^4^ Neuroimmunology and Neuromuscular Diseases Unit, Fondazione Istituto di Ricovero e Cura a Carattere Scientifico (IRCCS) Istituto Neurologico Carlo Besta, Milan, Italy; ^5^ Department of Human Neurosciences, Sapienza University of Rome, Rome, Italy; ^6^ Department of Medical-Surgical Sciences and of Biotechnologies, “Sapienza” University of Rome, Rome, Italy; ^7^ The Edmond and Lily Safra Center of Brain Science and The Life Sciences Institute, The Hebrew University of Jerusalem, Jerusalem, Israel; ^8^ Centre for Experimental Neurological Therapies (CENTERS), Department of Neurosciences, Mental Health and Sensory Organs, Sapienza University of Rome, Rome, Italy; ^9^ Bio@SNS Laboratory of Biology, Scuola Normale Superiore, Pisa, Italy

**Keywords:** multiple sclerosis, cladribine, ocrelizumab, DMT, biomarker, miRNA, PBMC

## Abstract

**Background and objectives:**

Multiple sclerosis (MS) is a chronic, progressive neurological disease characterized by early-stage neuroinflammation, neurodegeneration, and demyelination that involves a spectrum of heterogeneous clinical manifestations in terms of disease course and response to therapy. Even though several disease-modifying therapies (DMTs) are available to prevent MS-related brain damage—acting on the peripheral immune system with an indirect effect on MS lesions—individualizing therapy according to disease characteristics and prognostic factors is still an unmet need. Given that deregulated miRNAs have been proposed as diagnostic tools in neurodegenerative/neuroinflammatory diseases such as MS, we aimed to explore miRNA profiles as potential classifiers of the relapsing–remitting MS (RRMS) patients’ prospects to gain a more effective DMT choice and achieve a preferential drug response.

**Methods:**

A total of 25 adult patients with RRMS were enrolled in a cohort study, according to the latest McDonald criteria before (pre-cladribine, pre-CLA; pre-ocrelizumab, pre-OCRE, time T0) and after high-efficacy DMTs, time T1, 6 months post-CLA (*n* = 10, 7 F and 3 M, age 39.0 ± 7.5) or post-OCRE (*n* = 15, 10 F and 5 M, age 40.5 ± 10.4) treatment. A total of 15 age- and sex-matched healthy control subjects (9 F and 6 M, age 36.3 ± 3.0) were also selected. By using Agilent microarrays, we analyzed miRNA profiles from peripheral blood mononuclear cells (PBMC). miRNA–target networks were obtained by miRTargetLink, and Pearson’s correlation served to estimate the association between miRNAs and outcome clinical features.

**Results:**

First, the miRNA profiles of pre-CLA or pre-OCRE RRMS patients compared to healthy controls identified modulated miRNA patterns (40 and seven miRNAs, respectively). A direct comparison of the two pre-treatment groups at T0 and T1 revealed more pro-inflammatory patterns in the pre-CLA miRNA profiles. Moreover, both DMTs emerged as being capable of reverting some dysregulated miRNAs toward a protective phenotype. Both drug-dependent miRNA profiles and specific miRNAs, such as miR-199a-3p, miR-29b-3p, and miR-151a-3p, emerged as potentially involved in these drug-induced mechanisms. This enabled the selection of miRNAs correlated to clinical features and the related miRNA–mRNA network.

**Discussion:**

These data support the hypothesis of specific deregulated miRNAs as putative biomarkers in RRMS patients’ stratification and DMT drug response.

## Introduction

Multiple sclerosis (MS) is a chronic and progressive autoimmune demyelinating disease that affects the central nervous system (CNS). MS is characterized by a heterogeneous disease course and response to therapy (1–3). It is classified into three main phenotypes depending on the clinical disease course: relapsing–remitting (RRMS), displaying relapses and remission phases, and primary progressive MS characterized by gradual disability progression without acute relapses. Lastly, about 85% of patients initially diagnosed with RRMS shift toward a progressive stage called secondary progressive MS (SPMS) (4). People affected by MS accumulate progressive disability due to chronic inflammation and neurodegeneration which develop early in the disease process and are major drivers of progressive disability. It is generally felt that existing classifications of MS clinical course need improvements as they do not reflect the clinical and biological heterogeneity of the disease (5, 6).

Many disease-modifying therapies (DMTs) are now available to prevent the MS-related CNS damage (7, 8). Based on their efficacy, DMTs are identified as moderately (interferon-β dimethyl fumarate, etc.) and highly effective (ocrelizumab, OCRE; cladribine, CLA; etc.). Two high-efficacy DMTs were selected for the current study: CLA and OCRE. CLA is a synthetic purine analogue that induces apoptosis of B and T lymphocytes by the accumulation of intracellular chloro-deoxyadenosine triphosphate. In addition, it mediates immunomodulation in different immune cell populations. CLA tablets are administered in two short courses 1 year apart and reduce rapidly peripheral lymphocyte levels in patients with MS. Nadir is usually reached at week 9, and after that the lymphocyte counts gradually increase but remain lower than at baseline. OCRE (7, 9) is a humanized anti-CD20 monoclonal antibody which is administered intravenously every 6 months. It depletes CD20+ B cells in the blood to negligible levels after 14 days with a median time to B cell repletion of 72 weeks. Repopulation in CLA-treated patients is faster, with 91% of patients having at least 3% of B lymphocyte in the blood after 24 weeks compared to OCRE-treated patients (with 2% of patients having at least 3% of B lymphocyte after 23 weeks) (10–12). OCRE and CLA may act through mechanisms of action that, although different, could be both more “upstream” in the pathogenetic cascade with respect to others (e.g., they have a preponderant action on B cells, which may harbor an EBV infection which may have an etiologic relevance).

There is a high interindividual heterogeneity in the response patterns to DMTs. Identifying biological markers predictive of treatment response might allow the recognition of non-responders *versus* responders to drugs (13), enhancing the individualization of this therapeutic approach, respecting the individual patient’s needs, and optimizing healthcare costs. In this context, different miRNAs have been proposed as potential biomarkers of treatment response in neurodegenerative/neuroinflammatory diseases, such as MS (14–16). miRNAs are small non-coding RNAs that regulate gene expression at the post-transcriptional level, affecting several cellular processes, including inflammation, neurodegeneration, and remyelination by regulating different cellular processes (14). A large part of miRNAs are primate-specific, requiring human rather than murine studies (15). Many miRNAs are dysregulated in MS (16, 17). Specifically, studies have suggested that miRNAs are central in the functioning of T lymphocyte subtypes in MS and that their dysregulation may lead to a variation in the subtype balance (17, 18). Moreover, miRNAs play a regulatory role in maintaining proper B cell selection and activation (19).

Nowadays, the selection of specific DMTs is based on either the patient’s related factors such as disease-specific prognostic factors, comorbidities, risk tolerance, and pregnancy planning or drug-specific features such as efficacy and safety profile, route of administration, and treatment costs (20). Such criteria still need to be refined. The current study aimed to explore the use of miRNA profiles to improve patients’ stratification and potentially aid in treatment selection and efficacy.

Here we first compared two high-efficacy DMTs’ (CLA and OCRE) miRNA profiling in PBMCs from patients with RRMS. Initially, we compared the RRMS patients' miRNA levels to those of a healthy population as control (CTRs). Remarkable differences were highlighted in the miRNA profiles between the two subgroups previously selected for either treatment. The characterization of miRNA profiles before and after treatment, their correlation to clinical features (Frailty Index, FI; Expanded Disability Status Scale, EDSS) and the constitution of a specific miRNA–mRNA-related network allowed us to underpin differences in miRNA profiles under CLA or OCRE treatment and unveil specific miRNAs as potential biomarkers.

## Methods

### Patients’ selection and clinical evaluation for enrollment

A total of 25 adult patients with RRMS according to the latest McDonald revised criteria at the time of diagnosis (21) and followed at the Center for Multiple Sclerosis (Department of Human Neurosciences, Policlinico Umberto I—Sapienza University of Rome) were enrolled in a cohort study between 2021 and 2022. Furthermore, 15 healthy control subjects (age- and sex-matched) were selected. Furthermore, 10 RRMS patients received the first cycle of CLA tablets (1.75 mg/kg dose given in two treatment weeks of 5 days separated by 1 month), and 15 received the first course of OCRE (two 300-mg infusions at 2 weeks apart). DMTs have been chosen as clinically indicated (22). The patients were clinically tested before treatment initiation (pre-CLA/pre-OCRE – time 0 = T0) and 6 months after treatment (time 1 = T1). Sex, age at disease onset, FI, urinary symptoms, and EDSS were collected after the neurologist’s evaluation (**Table 1**). FI was calculated as the proportion of patient’s deficits based on a list of 42 binary items (23). To protect sensitive data, anonymous codes were assigned to each participant and preserved for the study duration. All subjects gave written informed consent to participate in the study. The research was conducted following the Helsinki Declaration and approved by the Ethics of Sapienza University—Policlinico Umberto I (Rif. 6361, protocol number 0635/2021). To minimize potential bias factors, all clinical data were collected in the same clinical center following the same guidelines.

**Table 1:** Demographic and clinical features in enrolled relapsing–remitting multiple sclerosis patients treated with cladribine (CLA) or ocrelizumab (OCRE).

Age	Sex	Disease duration at T1 (years)	EDSS T0	EDSS T1	FI T0	FI T1	Previous therapy	New lesions T0	GD+ enhancing lesions T0	Number of relapses in the last 12 months
A) CLA-treated patients										
36	F	1	1.5	1.5	0.10	0.10	Naïve	1	0	1
37	F	1	0	0	0.12	0.12	Naive	1	0	1
56	F	1	2	2	0.10	0.10	Naive	1	1	1
45	M	2	1.5	1.5	0.02	0.07	Naive	1	0	1
30	M	2	0	0	0.02	0.02	Naive	1	1	1
38	M	2	4	4	0.19	0.19	Naive	1	1	1
31	F	3	1	1	0.17	0.17	Switched	1	1	1
36	F	9	1.5	1.5	0.05	0.05	Switched	1	1	1
38	F	14	2	2.5	0.19	0.19	Switched	0	0	1
43	F	21	1	1	0.12	0.12	Switched	1	1	1
B) OCRE-treated patients										
36	F	1	1	1	0.02	0.02	Naïve	1	0	1
23	M	1	2	1	0.05	0.05	Naive	1	0	1
56	M	2	2	2	0.26	0.26	Naive	1	1	1
36	F	3	2.5	2.5	0.24	0.24	Naive	1	1	1
41	M	15	2.5	1.5	0.07	0.07	Naive	1	0	1
52	M	2	5.5	1.5	0.19	0.19	Switched	0	0	1
32	F	3	1.5	1	0.07	0.07	Switched	1	1	1
37	F	3	1.5	1.5	0.05	0.05	Switched	1	1	1
44	F	3	1	1	0.07	0.07	Switched	1	0	1
28	F	6	1.5	1.5	0.12	0.12	Switched	1	1	1
43	F	15	2	1.5	0.12	0.12	Switched	0	0	1
44	F	18	5.5	6	0.19	0.19	Switched	1	0	1
60	M	26	7	7	0.14	0.14	Switched	1	0	1
46	F	27	2	2	0.07	0.07	Switched	1	0	1
30	F	7	2	2	0.05	0.05	Switched	1	0	1

### Blood sample and PBMC collection

The patients’ peripheral blood was collected by venipuncture at T0 and T1 for miRNAs’ profiling and other laboratory tests. The blood samples for both patients and healthy subjects were collected in Vacutainer tubes containing EDTA. Then, 15 ml of phosphate-buffered saline (PBS; without Ca^2+^, Mg^2+^) was added to 10 ml of each samples’ blood; after mixing, the diluted blood samples were carefully layered onto 7.5 ml of Ficoll for 30 min of centrifugation (18–20°C) at 1,800 rpm. The tubes exhibited four different layers containing plasma, lymphocytes, and monocytes, Ficoll, granulocytes, and erythrocytes, respectively, from top to bottom. The lymphocytes/monocytes layer was accurately collected in clean tubes; the cells were then pelleted (1,400 rpm for 10 min at 18–20°C) and washed with PBS. The dry pellet was finally stored at -80°C.

### RNA extraction and quality control

RNA extraction was performed according to the miRNeasy Tissue/Cells Advanced Mini Kit (QIAGEN^©^) instructions: the cells were suspended in 500 μL of RTL buffer + β mercaptoethanol, incubated at 37°C for 10 min, and homogenized. The samples were passed through two different spin columns: the gDNA Eliminator spin column to remove all DNA and the Rneasy spin column to select RNA molecules. The miRNeasy Tissue/Cells Advanced Kits enabled efficient RNA enrichment down to approximately 18 nucleotides in size. All RNA samples were again stored at -80°C. RNA purity and concentration quality control included the evaluation of absorbance at 260 nm by NanoDrop ND-1000 (Labtech International, Ringmer, UK). To assess the RNA integrity, samples were tested in the Agilent 2100 Bioanalyzer (Agilent Technologies, Santa Clara, CA, USA) *via* the Eukaryote Total RNA 6000 Nano kit (Agilent Technologies, Santa Clara, CA, USA) and the Small RNA kit (Agilent Technologies, Santa Clara, CA, USA). The bioanalyzer assessed each sample’s RNA integrity number (RIN). Samples displaying an under-threshold RIN value <8.0 were excluded.

### miRNA profiles

miRNA profiles were performed according to the standard “Agilent miRNA Microarray System, miRNA Complete Labelling, and Hyb Kit” protocol (Agilent Technologies, Version 3.1.1, 2015). After a phosphatase treatment and a denaturation process *via* DMSO, 100 ng of RNA, extracted from each sample, was labeled with 3-pCp cyanine. The samples were then dried through a vacuum concentrator, and a 45-µl hybridization solution was added to each one. Then, the samples were hybridized to the Agilent Human miRNA Microarrays chip 8x60K (Agilent PN G4870-60530, grid ID = 070156) containing 2,549 human miRNAs. The glasses were incubated in the Agilent Hybridization Oven at 55°C, 10 RPM, for 20 h, washed according to the protocol, and scanned using the Agilent DNA Microarray Scanner (G2539C).

### Statistical analysis

miRNAs’ profiling was assessed by using Agilent Platform (Agilent Technologies, Milan, Italy). The samples were processed according to Agilent’s standard experimental procedure. Data analysis was performed using R-Bioconductor (Seattle, WA, USA). The samples were log_2_-transformed and normalized by the median alignment method; differentially expressed (DE) miRNAs were selected by the R-limma tool with threshold false discovery rate <0.05 and linear |fold change| >1.5 (24). Possible confounding factors such as sex (M/F) and previous therapy (yes/no) were included in limma linear models as covariates. Only miRNAs with expression values >0.0 in every sample were included in the analysis. Pearson’s correlation coefficient was used to estimate the association between miRNA profiles and clinical measures. Interaction miRNA–target networks were obtained using miRTargetLink 2.0, selecting only validated connections and miRNAs with >4 targets, and further processed through using Cytoscape. Clinical information was compared using either Mann–Whitney or Fisher’s exact test according to data type.

The manuscript preparation followed the STROBE cohort reporting guidelines (25).

### Data availability statement

Anonymized data not published within this article will be made available upon request from any qualified investigator. Access to anonymized individual patient's data, including raw datasets, analysis-ready datasets, statistical analysis plans, clinical data, and dataset specifications, may be provided upon request of any qualified investigator. miRNAs’ complete raw and processed data are freely available from the Gene Expression Omnibus database with accession number GSE230064.

## Results

### Multiple sclerosis miRNAs’ profiling

A total of 25 adult patients with RRMS were enrolled in a cohort study according to the latest McDonald criteria (21) before (pre-CLA; pre-OCRE, time T0) and after high-efficacy DMTs, time T1, 6 months post-CLA (*n* = 10, 7 F and 3 M, age 39.0 ± 7.5) and post-OCRE (*n* = 15, 10 F and 5 M, age 40.5 ± 10.4) treatments. The demographic and clinical features of the CLA- or OCRE-treated RRMS patients are reported in **Table 1** and the statistical comparisons in **Supplementary Table S5**.

A total of fifteen healthy control subjects who were age- and sex-matched (9 F and 6 M, age 36.3 ± 3.0) were selected. All 25 RRMS patients had completed follow-up 6 months after specific treatment. miRNAs’ profiling was performed by microarray analysis on PBMCs from whole blood. To identify specific modulated miRNAs, the following comparisons were analyzed: pre-CLA vs. CTRs, pre-OCRE vs. CTRs, pre-CLA vs. pre-OCRE, CLA post-treatment (T1) vs. pre-CLA (T0), and OCRE post-treatment (T1) vs. pre-OCRE (T0).

### Comparison between pre-treatment patients and controls

**Figure 1:**
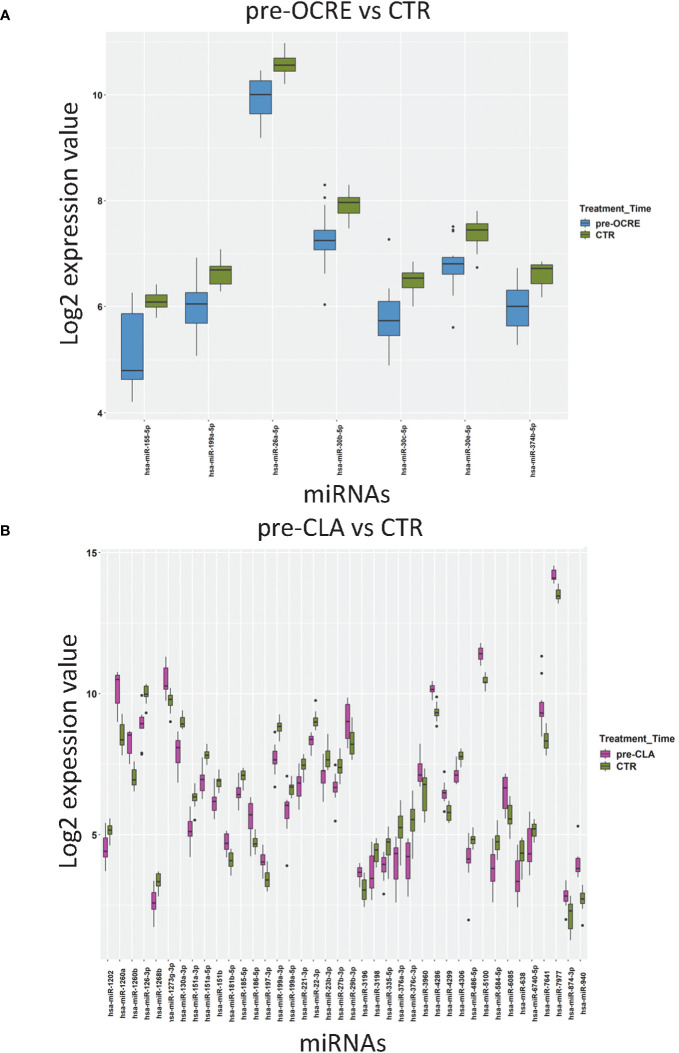
Relapsing–remitting multiple sclerosis patients, clinically selected for cladribine (CLA) or ocrelizumab (OCRE) treatment, display different miRNA profiles. **(A)** Pre-OCRE (*n* = 15) vs. CTRs (*n* = 15) comparison. **(B)** Pre-CLA (*n* = 10) vs. CTRs (*n* = 15) comparison. Log_2_ median normalized values of differentially expressed miRNAs were selected using the following thresholds: R limma test false discovery rate <0.05 + linear |fold change| >1.5. The boxes correspond to median ± interquartile range, and the black dots are outliers.

We identified a final list of 200 miRNAs expressed in every sample. The patients’ miRNA profiling preceding CLA (pre-CLA) and OCRE treatment (pre-OCRE), compared to CTRs, was analyzed and reported in **Figures 1A, B**. A total of 40 miRNAs resulted to be deregulated, of which 17 were upregulated (among which were miR-181b-5p, miR-186-5p, miR-486-5p, and miR-29b-3p) and 23 were downregulated (including miR-151a-3p, miR-151a-5p, miR-23b-3p, miR-27b-3p, and miR-199a-3p) when comparing pre-CLA-treated patients to CTRs (**Figure 1B**). Conversely, seven miRNAs were downregulated (miR-155-5p, miR-199a-5p, miR-26a-5p, miR-30b-5p, miR-30c-5p, miR-30e-5p, and miR-374-5p) in the pre-OCRE vs. CTRs comparison (**Figure 1A**). These results highlight how the RRMS patients displayed deregulated miRNA profiles compared to healthy controls.

### MS patients’ stratification and response to specific DMTs (CLA or OCRE) according to the miRNA profiles

**Figure 2:**
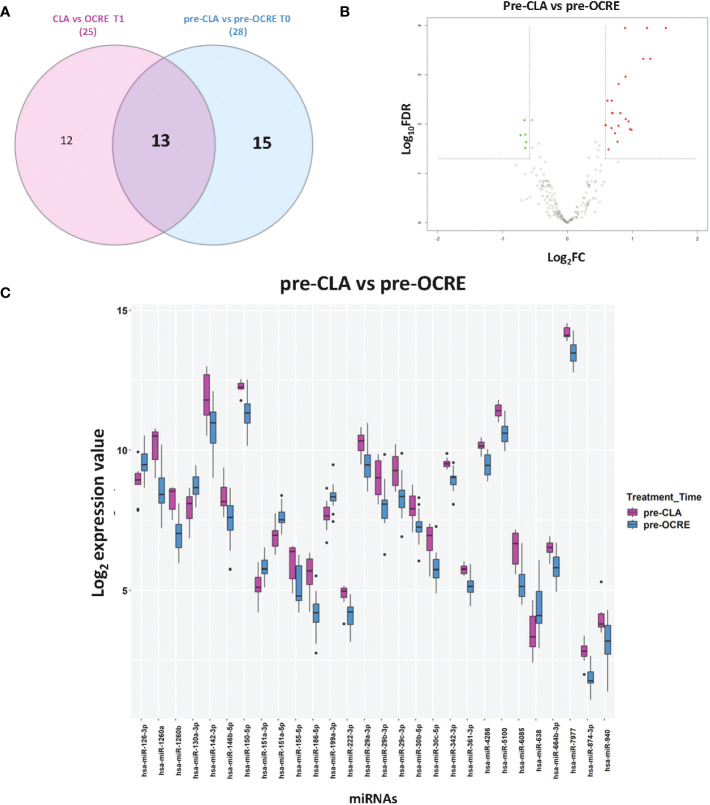
Direct comparison of pre-cladribine (CLA) and pre-ocrelizumab (OCRE) miRNA profiles. **(A)** Venn diagram for the differentially expressed (DE) miRNAs between groups of patients preceding either CLA (pre-CLA) or OCRE (pre-OCRE) treatment or CLA at T1 vs. OCRE at T1. **(B)** Volcano plot of the pre-CLA (*n* = 10) vs. the pre-OCRE (*n* = 15) miRNAs. DE miRNAs (green: downregulated; red: upregulated) are shown. **(C)** Levels of DE miRNAs in pre-CLA vs. pre-OCRE. Log2 median normalized values of DE miRNAs are shown; miRNAs were selected using R limma test false discovery rate <0.05 + linear |fold change| >1.5 thresholds. The boxes correspond to median ± interquartile range, and the black dots are outliers.

The miRNA profiles were compared between the two groups of patients before (pre-CLA T0 vs. pre-OCRE T0) and 6 months after treatment (CLA T1 vs. OCRE T1) to explore differences between baseline and treatment-specific response. The volcano plot resulting from the pre-CLA vs. the pre-OCRE miRNAs’ comparison exhibited (**Figure 2B**) 28 DE miRNAs: 22 upregulated miRNAs (out of which the most upregulated were miR-1260a, miR-1260b, miR-186-5p, miR-6085, miR-142-3p, miR-29b-3p, miR-29c-3p, and miR-155-5p) and six downregulated ones (miR-638, miR-130a-3p, miR-151a-5p, miR-126-3p, miR-151a-3p, and miR-199a-3p) (15 + 13 in the Venn diagram, **Figure 2A**), whose main functions are described in **Supplementary Table S1**, according to the cited literature.

The Venn diagram shows the DE miRNAs shared between patients preceding CLA (pre-CLA) or OCRE (pre-OCRE) treatment (**Figure 2A**) and between the same subjects analyzed 6 months post-treatment: (CLA T1 vs. OCRE T1) (**Supplementary Figure S1**). The log_2_ median expression values of the DE miRNAs in the pre-CLA vs. the pre-OCRE comparison are shown in **Figure 2C**. Furthermore, the data indicated 25 DE miRNAs in the CLA T1 vs. OCRE T1 comparison, with five downregulated and 20 upregulated (**Supplementary Figure S1**).

Literature annotation is reported in **Supplementary Table S2**. The differential miRNAs’ profiling underlined a higher expression of inflammation-related miRNAs in patients selected for CLA treatment (pre-CLA) compared to patients selected for OCRE treatment (pre-OCRE). These dysregulated miRNAs are known to be involved in inflammatory and immune pathways (**Supplementary Table S1**), including miR-155-5p, which is highly pro-inflammatory, and the upregulated miR-186-5p, over-expressed in MS while downregulated in other neurodegenerative diseases and aging.

**Figure 3:**
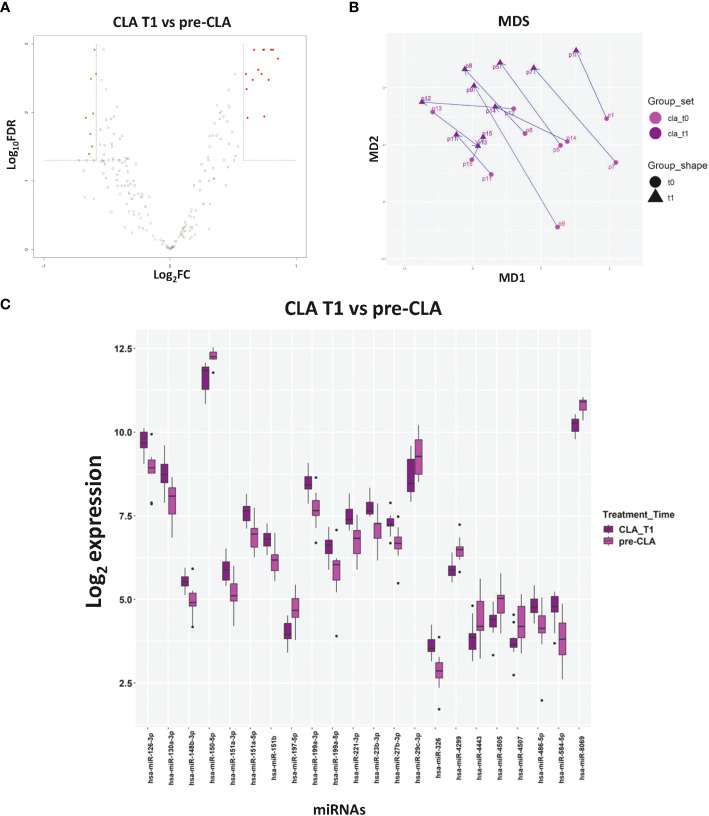
The impact of cladribine (CLA) treatment on miRNA profiles. **(A)** Volcano plot of the CLA T1 (*n* = 10) vs. pre-CLA (*n* = 10) miRNAs. **(B)** Multidimensional scaling of CLA samples based on the miRNA profiles. Each arrow corresponds to a different patient treated with CLA and connects the miRNA profile at T0 (light purple dot) to the profile at T1 (dark purple dot). **(C)** Levels of differentially expressed (DE) miRNAs in CLA T1 vs. pre-CLA. Log2 median normalized values of DE miRNA are shown. The CLA T1 samples correspond to subjects with relapsing–remitting multiple sclerosis, whose miRNA levels were analyzed 6 months after CLA treatment initiation. miRNAs were selected using R limma test false discovery rate <0.05 + linear |fold change| >1.5 thresholds. The boxes correspond to median ± interquartile range, and the black dots are outliers.

The multidimensional scaling plot of miRNA profiles in **Figure 3B** displayed how most of the patients had similar miRNA responses to CLA treatment, as suggested by the largely parallel direction of the T0 to T1 trajectories, while the response to OCRE (**Supplementary Figure S2**) was more heterogeneous. To unveil the impact of the selected DMT on the enrolled RRMS patients, we compared the miRNA profiles 6 months after CLA or OCRE treatment to the pre-treatment levels. A total of 22 miRNAs resulted to be modulated by CLA (**Figures 3A, B**) (14 upregulated: miR-151a-3p, miR-151a-5p, miR-130a-3p, miR-126-3p, miR-151b, miR-326, miR-584-5p, miR-1991a-3p, miR-199a-5p, miR-23b-3p, miR-221-3p, miR-27b-3p, miR-486-5p, and miR-148b-3p and eight downregulated: miR-150-5p, miR-197-5p, miR-29c-3p, miR-4299, miR-4443, miR-4505, miR-4507, and miR-8069), while only one was modulated by OCRE treatment (**Supplementary Figure S2**). Concerning the differential CLA profiling, most DE miRNAs were involved in MS, as described in the literature mentioned in **Supplementary Table S3**. Specifically, some of the DE responding miRNAs (miR-199a-3p, miR-199a-5p, miR-29b-3p, and miR-23b-3p), whose levels were modulated by the CLA treatment, are known to be dysregulated and of clinical interest in MS. In comparison, the OCRE treatment modulated only miR-3653-3p (**Supplementary Figure S2**), which resulted to be downregulated and is known to be involved in carcinogenesis and glioma progression and other carcinoma diffusions.

### Associations between clinical measures and DE miRNAs

**Figure 4:**
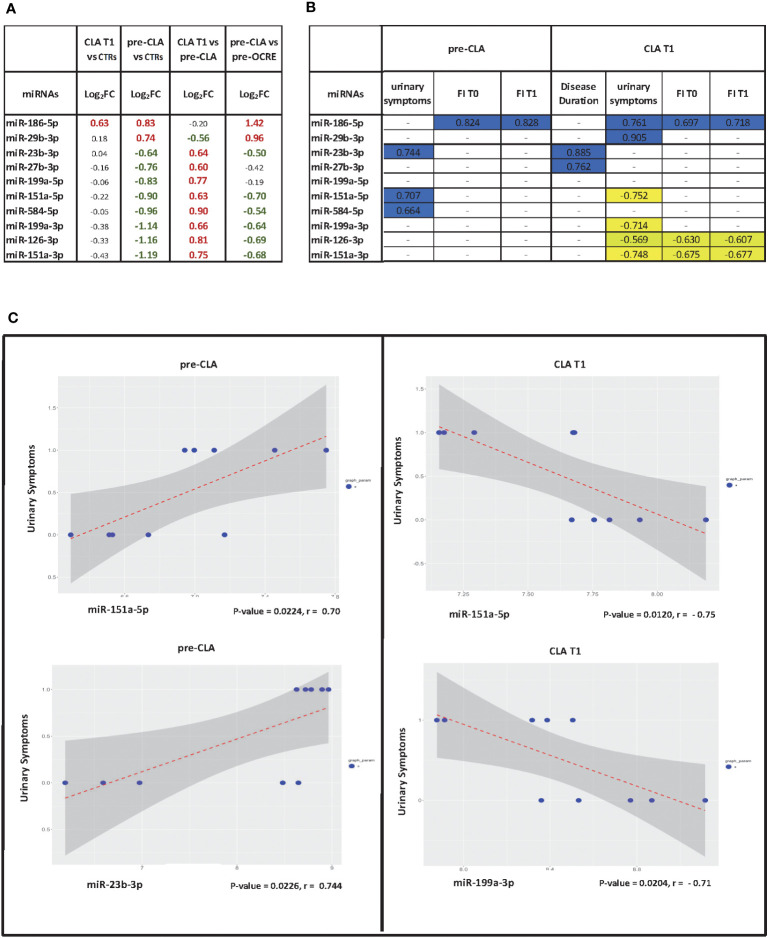
Pearson correlations between miRNA levels and clinical data. **(A)** Pearson’s correlation index between differentially expressed miRNAs (cladribine (CLA) T1 vs. pre-CLA) and selected statistically significant clinical variables. T1 = 6 months after the treatment’s first administration/infusion. Analyzed clinical variables: urinary symptoms (yes/no, binary); EDSS score (integer discrete); FI score; disease duration in months. **(B)** Table of significant correlations. **(C)** Scatter plot of expression levels vs. clinical variable for selected correlations with miRNA in the pre-CLA and CLA T1 groups; the regression line is drawn.

The Pearson’s analysis between DE miRNAs and clinical data in the pre- and post-CLA treatment groups showed miRNAs which were significantly correlated to the FI (miR-186-5p), disease duration (miR-23b-3p and miR-27b-3p), or urinary symptoms (miR-186-5p, miR-29b-3p, miR-151a-5p, miR-199a-3p, and miR-151-3p), as reported in **Figures 4A–C**. Other analyses are reported in **Supplementary Figures S3–S5**. We then compared the dysregulated miRNAs associated with MS, according to the human miRNA Disease Database and the miR2 Disease Database (26), to the clinical features. A total of 10 miRNAs were selected for further analysis (miR-126-3p, miR-186-5p, miR-29b-3p, miR-23b-3p, miR-27b-3p, miR-199a-3p, miR-199a-5p, miR-151a-3p, miR-151a-5p, and miR-584-5p). Eight out of 10 were significantly upregulated in the CLA T1 vs. pre-CLA comparison while downregulated in the pre-CLA vs. CTRs comparison (**Figure 4A**). The miRNAs also presented a few opposite clinical correlation profiles when comparing pre-CLA to post-CLA treatment. In particular, specific miRNAs shifted from being directly correlated to a significant inverse correlation to urinary symptoms (**Figures 4B, C**).

### miRNA target network

**Figure 5:**
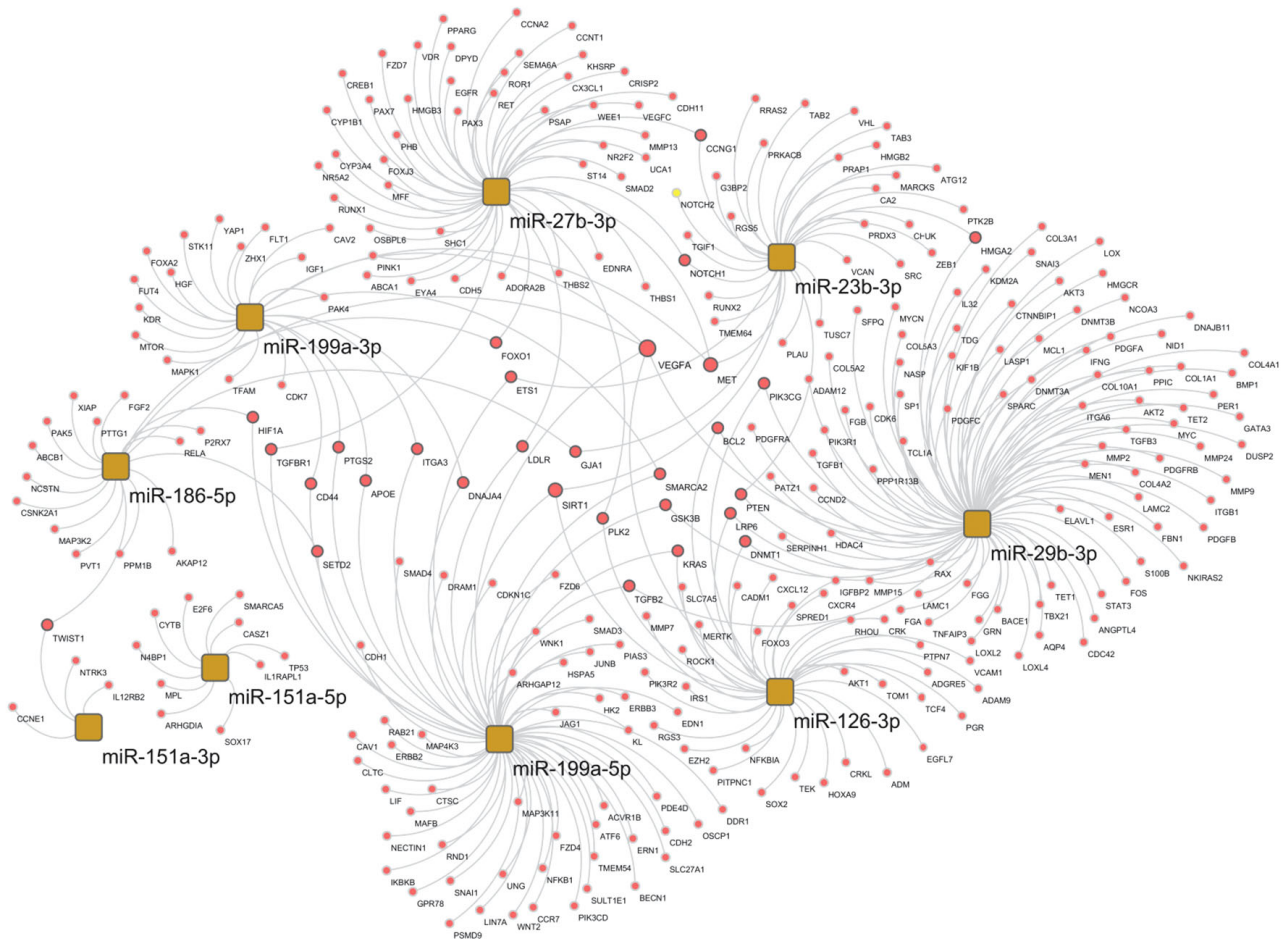
Selected miRNA–target network in the cladribine (CLA) T1 vs. pre-CLA T0 comparison. miRNA–target network of selected miRNAs in the CLA T1 vs. pre-CLA T0 comparison obtained by the miRTargetLink 2.0 tool, selecting only validated mRNA targets and miRNAs with >4 targets, and further processed through Cytoscape.

**Figure 6:**
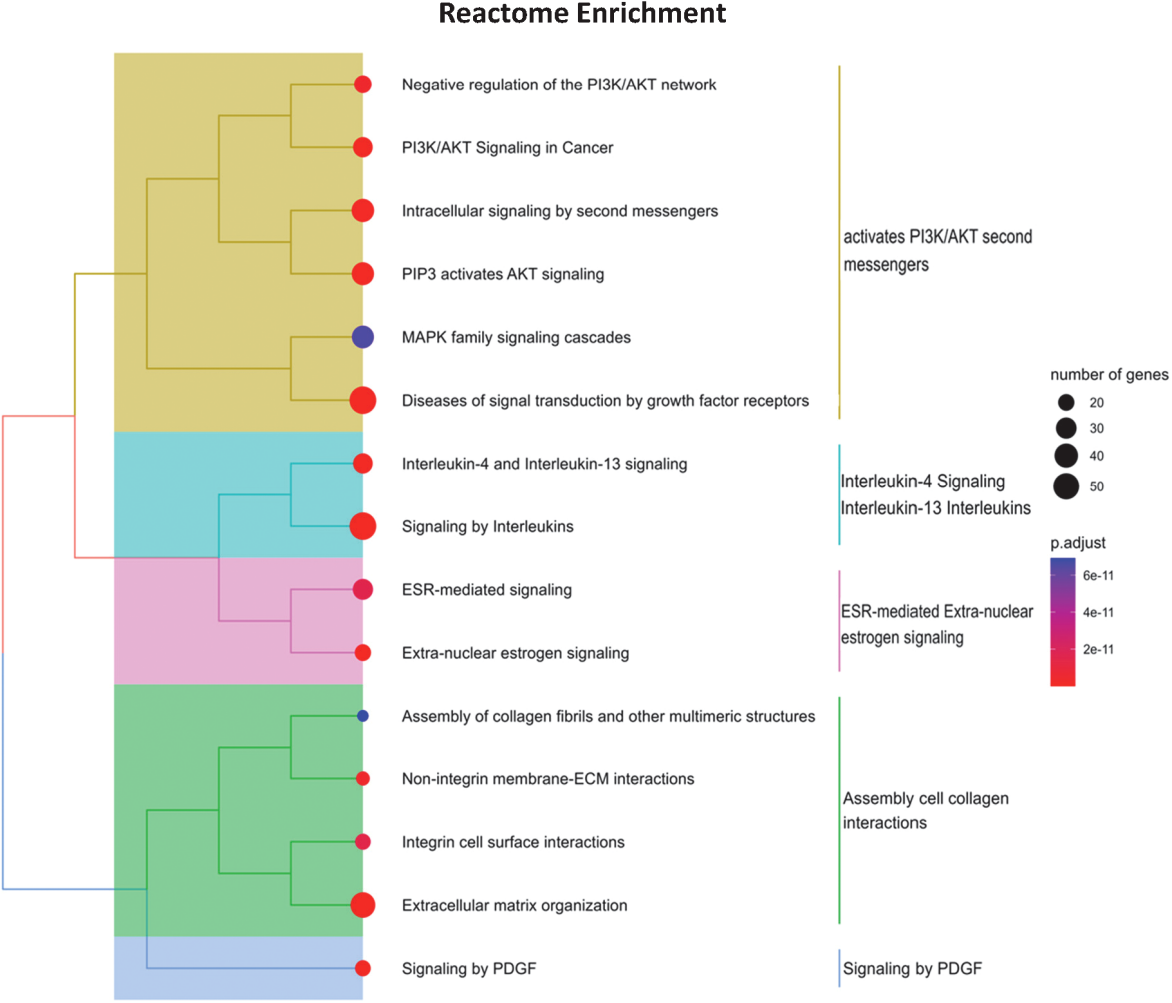
Pathway enrichment analysis of miRNA gene targets. The dendrogram is a hierarchical clustering of enriched Reactome pathways, using the gene lists overlap as distance metric. The pathways are further clustered into broader functional categories. The dot size is proportional to the gene list size, while the dot color corresponds to the false discovery rate adjusted *p*-value of enriched terms. The analyzed gene list was selected by the mirTargetLink predictor, using only strong validated targets. The plot was obtained by the clusterProfiler R package.

We analyzed the mRNA–miRNA network to assess the effect of the 10 selected miRNAs whose expression levels were reverted by the specific DMT (CLA) and were also correlated to clinical features (**Figure 5**). The network comprises 335 validated edges between the nine miRNAs (miR-126-3p, miR-186-5p, miR-29b-3p, miR-23b-3p, miR-27b-3p, miR-199a-3p, miR-199a-5p, miR-151a-3p, and miR-151a-5p, with miR-584-5p excluded as presenting no strong interactions to targets) and mRNA targets. Out of the 335 validated edges, 272 were single connections, 26 genes are targets shared by two miRNAs, MET and SIRT1 are targets shared by three miRNAs (miR-199a-3p, miR-27b-3p, and miR-23b-3p as well as miR-126-3p, miR-199a-5p, and miR-23b-3p, respectively), and lastly VEGF-α is a predicted target for 5 miRNAs (miR-29b-3p, miR-126-3p, miR-186-5p and 199a-3p and miR-199a-5p). To better outline the cellular functions involved in the produced network (**Figure 5**), a pathway enrichment analysis was generated (**Figure 6**). Most of the specific genes in the network are involved in pathways including processes concerning interleukins 4 and 13 signaling, PIK3T/AKT second messengers, collagen, extra-nuclear estrogen signaling and signaling by PDGF, inflammation, angiogenesis, and blood–brain barrier integrity; others appeared to present a neuroprotective role as shown in the pathway dendrogram (**Figure 6**).

## Discussion

The present cohort study investigated miRNA profiles in PBMCs collected from RRMS patients before and after treatment with CLA or OCRE. Since miRNAs are increasingly considered as biomarkers in multiple neurodegenerative/neuroinflammatory diseases, we first compared the miRNA profiles of patients who were clinically selected for either CLA or OCRE treatment to healthy controls (pre-CLA vs. CTRs or pre-OCRE vs. CTRs). The analysis identified some dysregulated miRNAs possibly involved in MS pathogenesis and specifically characterized each group. Among the 40 DE miRNAs (pre-CLA vs. CTRs), several are notably involved in inflammatory and immune pathways, such as miR-23b-3p (27), the downregulated miR-151-5p (28, 29) and miR-151-3p (26), miR-27b (30), and miR-29b, contributing to the regulation of Th1 and Th17 differentiation (31). In line with part of the current literature (32), we found a downregulation of miR-126-3p which, however, has also been reported as increased in RRMS patients (33). Furthermore, miR-126-3p, expressed in the CNS endothelium, displays a possible contribution to the regulation of leukocyte adhesion to human brain endothelium (34), a critical step in triggering the blood–brain barrier’s (BBB) damage process. Thus, we suggest that these miRNAs may influence the inflammatory process and play a role in the MS patients’ BBB disruption.

The pre-OCRE miRNAs’ profiling (*vs* CTRs) is characterized by a significant downregulation of miR**-**155-5p (**Figure 1**), known as *inflamma-miR*, which influences myeloid cell polarization, leading to a pro-inflammatory phenotype (35). miR-155-5p also disrupts the BBB *via* key junctional proteins under inflammatory conditions. It drives demyelination processes by contributing to microglial activation, the polarization of astrocytes, and the downregulation of CD47 protein and affecting crucial transcription factors.

miR-155-5p has been reported as downregulated exclusively in SPMS patients (36). Since OCRE is used in patients with highly active MS and due to the current lack of prognostic factors for long-term disability (i.e., at a higher risk for neurodegenerative mechanisms), our data regarding the downregulation of miR-155-5p in OCRE groups propose miRNA profiles as possible prognostic factors in the disease progression.

The only DE miRNA, shared by both pre-CLA and pre-OCRE populations when compared with CTRs, is the downregulated miR-199a-5p, which negatively correlates with EDSS in MS (18). Together with other miRNAs, miR-199a-5p may participate in the control of the balance between innate and adaptive immunity, avoid chronic inflammation, and prevent neuroinflammation. The imbalance between pro- and anti-inflammatory processes may modify the immune activities at the glia level, leading to neuronal damage and altering BBB’s integrity (37).

In addition to analyzing differences in the pre-treatment profiles, we directly compared the two miRNA profiles (pre-CLA vs. pre-OCRE). This comparison confirmed that the modulated miRNAs were involved in inflammatory and immune pathways (**Supplementary Table S1**), including relevant upregulated genes such as miR-155-5p and miR-186-5p, which are downregulated in elderly people and those with a neurodegenerative disease (26, 38). The upregulation of miR-30c, previously implicated in MS (39) and characterized in response to pharmacological treatments (19, 40), together with the downregulation of miR-199a in the MS relapsing phase is correlated with more frequent Th17 cell differentiation and the severity of the disease (41). These data suggest a more inflammatory profiling of the patients selected for CLA compared to the pre-OCRE ones, supporting the possible use of miRNAs for a personalized DMT choice.

When we compared the expression profiles in samples obtained during the two treatment courses, we found that CLA produced a higher impact on DE miRNAs than OCRE, i.e., CLA modulated a higher number of miRNAs (22 vs. one miRNA), mainly involving immune and inflammatory targets. This is also suggested by the clinical trajectories (from T0 to T1) in multidimensional scaling plots: CLA trajectories (**Figure 3**) are largely parallel, with T0 and T1 sets clearly separated, while under OCRE treatment (**Supplementary S2**) the T0 and T1 sets are widely overlapping. The homogeneous response in the multidimensional scaling plot of CLA patients compared to the heterogeneous one of OCRE patients can be related to the different mechanism of action of the DMTs or to the different kinetic of cell depletion and reconstitution. It may also reflect an immunomodulatory effect of CLA on the immune system. However, further studies of miRNA profiles in different lymphocyte subsets should be performed.

Anyway, no statistically significant differences were found between the two groups regarding EDSS, duration of disease, number of relapses, presence of new lesions, or presence of gadolinium-enhancing lesions on magnetic resonance imaging (MRI) at T0 and the number of relapses in the last 6 months (**Supplementary Table S5**). A wider number of patients and a longer follow-up might be necessary to identify clinical changes.

Notably, miR-3653-3p is involved in carcinogenesis and glioma progression and other carcinoma diffusions. Correspondingly, the OCRE effect may be related to its direct long-term depletion of memory B cells (42) or through the T cell activity blockade (43). Furthermore, most CLA-treated patients maintained B cell levels of at least 1% of total lymphocyte count and recovered to at least 10–20 circulating CD19 B cells/μL at 24 weeks after treatment, contrasting with prolonged B cell depletion detected after OCRE (11). Importantly, nevertheless, both DMTs successfully reverted some dysregulated miRNA profiles toward a protective phenotype. Our analysis exhibited baseline DE values of specific miRNAs (miR-199a-3p, miR-199a-5p, miR-29b-3p, and miR-23b-3p) whose expression is reversed by CLA. As discussed above, miR-199a-3p and 199-5p upregulation, together with the dysregulation of miR-23b-3p, miR-151a-5p, and miR-151a-3p, are directed toward an anti-inflammatory and neuroprotective phenotype. Pearson’s correlations allowed the identification of miRNAs that better correlate to some clinical features, such as urinary symptoms, FI, or EDSS. Additionally, it has been estimated that 80%−90% of patients living with MS suffer from some form of lower urinary tract symptoms over the course of the disease, representing an important challenge for both patients and caregivers (44). Correspondingly, some miRNAs present opposite clinical correlation profiles when comparing the pre-CLA and post-CLA treatments. Thus, both miR-23b-3p and miR-199a-3p, which correlated to urinary symptoms, have been reported as downregulated in MS inflammatory lesions, which was compatible to our findings in the pre-CLA profiling, and were then reverted by the CLA treatment. Interestingly, specific miRNAs whose levels correlated to urinary symptoms shifted from direct to a significantly inverse correlation (**Figures 4A, B**). It would be interesting to monitor these clinical features over time to reveal whether these correlations, which shift from direct to inverse, correspond to symptom improvement and better prognosis.

To better outline the different pathways involved in the mRNAs–miRNA–target network (**Figure 5**), a pathway enrichment analysis was generated (**Figure 6**) and revealed potential mechanisms linking neuroinflammation and neurodegeneration to CLA treatment. Briefly, interleukins 4 and 13 signaling, PIK3T/AKT second messengers, collagen, extra-nuclear estrogen signaling, and signaling by PDGF emerged as functionally involved in CLA-modulated miRNA pathways. Both the closely related interleukin (IL)-13, T cell-derived cytokine, and Il-4 routes play powerful anti-inflammatory roles in MS (45). Concerning the extracellular matrix (ECM) members in MS, such as collagen, we propose that these miRNAs control the inflammatory response and treatment induces repair since the crosstalk between the ECM and immune responses (IL-4 and Il-13) is known to modulate lesions for recovery or worsening (46). Furthermore, the link with estrogen nuclear signaling in MS might mediate anti-inflammatory cytokine production and Treg cell expansion (47).

We also evinced that platelet-derived growth factor (PDGF) signaling is involved in the network modulated by miR-29b-3p (which is downregulated by the CLA treatment). PDGF plays a substantial role in long-term potentiation (LTP) and brain reserve in MS patients, as this molecule is associated with more pronounced LTP in RRMS patients and with the compensation of new brain lesions in RRMS (48). The brain plasticity reserve, in the form of LTP, is crucial to contrast clinical deterioration in MS. Enhancing PDGF signaling might represent a valuable treatment option to slow disease progression by maintaining brain reserve and reducing neuronal damage (49).

The tightly connected genes in the abovementioned network (**Figure 5**) further include the vascular endothelial growth factor alpha (VEGF-α), known to be involved in inflammation, angiogenesis (50), and BBB integrity with a crucial role in MS development and progression. VEGF-α plays a major role in the development of neurodegenerative diseases through inflammation, first acting as a pro-inflammatory initiator but later leading to lower responsivity of angiogenic molecules. On one hand, significant correlations were reported between VEGF-α levels and BBB disruption in MS plaques. On the other hand, decreased levels of VEGF-α have been reported in MS patients’ CSF (51, 52). Other network genes include Sirtuin1 (SIRT1) and hepatocyte growth factor receptor (MET). SIRT1 was found to be elevated in MS brain lesions and in cells from both acute and chronic active MS lesions (53). However, altered SIRT1 expression and activity have also been linked to inflammatory diseases. MET activation is involved in angiogenesis, wound healing, cell scattering, proliferation, and cancer invasion (54) and might exert neuroprotective and immunomodulatory effects in the experimental allergic encephalomyelitis model (EAE) of MS.

Between the miRNAs reverted by the CLA treatment, we discussed relevant candidates including miR-199a, miR-151a-5p, and miR-29b-3p. miR-199a, which is downregulated in the pre-CLA vs. the CTRs and upregulated by CLA, is involved in Treg differentiation (41). miR-151a-5p, the most widely modulated miRNA by CLA treatment, is not yet characterized in MS pathophysiology and needs to be further explored, and miR-29b-3p, the only downregulated miRNA after CLA treatment, is strongly connected to the PDGF pathway as discussed above.

In conclusion, we identified drug-dependent changes in miRNA profiles and identified potential candidate miRNAs that we propose to be involved in the corresponding pharmacological mechanisms.

The strength of this study is the identification of DE miRNAs as potential biomarkers in RRMS patients’ stratification and of CLA or OCRE drug response. The limitations of the study include the limited number of participants, duration of the follow-up, and the fact that not all patients are naïve to DMTs. Thus, further studies on a wider population and independent cohorts monitored for longer time periods are needed. Moreover, small RNA-Seq analysis could extend our findings, allowing the identification of other short non-coding RNAs potentially involved in MS pathogenesis, monitoring, and therapeutic response.

## Data availability statement

The datasets presented in this study can be found in online repositories. The names of the repository/repositories and accession number(s) can be found below: https://www.ncbi.nlm.nih.gov/geo/, GSE230064.

## Ethics statement

The studies involving humans were approved by Ethics of Sapienza University – Policlinico Umberto I. The studies were conducted in accordance with the local legislation and institutional requirements. The participants provided their written informed consent to participate in this study.

## Author contributions

IA: including medical writing, study concept or design, analysis or data interpretation, and statistical analysis. LM: including medical writing, major role in data acquisition, study concept or design, and analysis or data interpretation. VM and RB: major role in data acquisition, RNA extraction and qualitative analysis, and microarray analysis. TS: major role in data acquisition and RNA extraction and microarray analysis. CD’A: major role in the acquisition of data, RNA extraction and qualitative analysis, and microarray analysis. SC and GF: major role in data acquisition and critical reading of the manuscript. DB: including medical writing and analysis or interpretation of data. FM and RF: analysis or interpretation of data and revision of the manuscript. EP: including medical writing and major role in data acquisition. HS: analysis or data interpretation and revision of the manuscript. MS: including medical writing and analysis or data interpretation. ACa: analysis or data interpretation. MD’O: including medical writing, study concept or design, and analysis or data interpretation. ACo: including medical writing for content and major role in data acquisition, study concept or design, and analysis or data interpretation. All authors contributed to the article and approved the submitted version.
