# Supplementary material for: Cladribine and ocrelizumab induce differential miRNA profiles in peripheral blood mononucleated cells from relapsing–remitting multiple sclerosis patients
Source: Front Immunol. 2023 Dec 13;14:1234869. doi: 10.3389/fimmu.2023.1234869 (PMC10751352; doi:10.3389/fimmu.2023.1234869)

Supplementary Figure S1

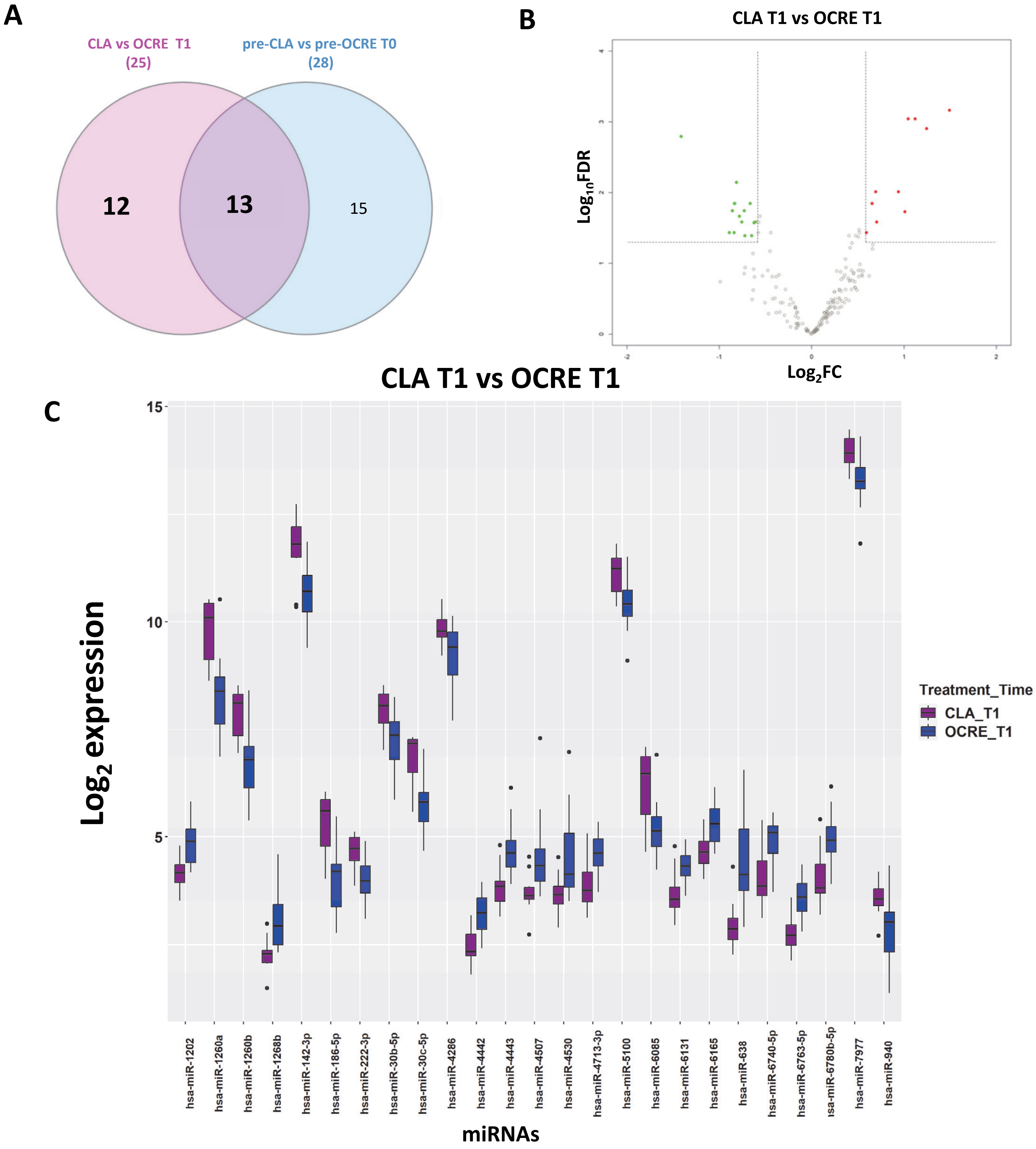

Supplementary Figure S2

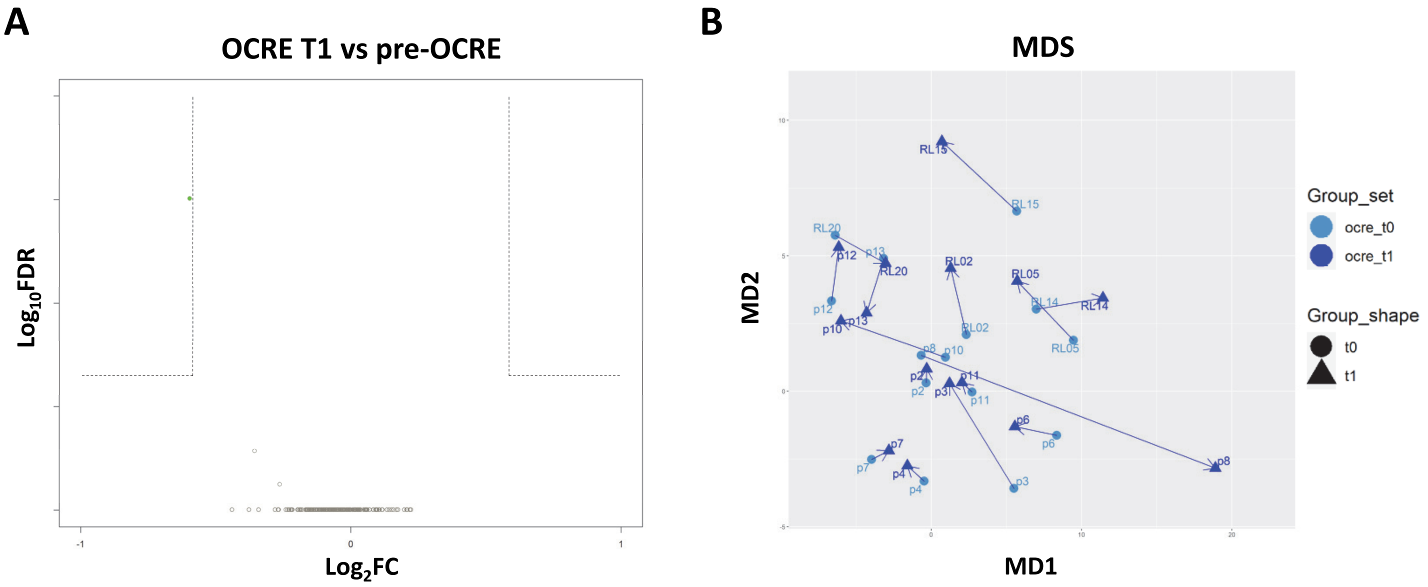

Supplementary Figure S3

	log_2FC (PRE-CLA VS PRE-OCRE)	PRE-CLA					PRE-OCRE			
		Urinary symptoms	EDSS T0	FI T0	EDSS T1	FI T1	Disease Duration at T0	Urinary symptoms	FI T0	FI T1
miR-1260a	1.52	-	-	0.77	-	0.81	-	-0.71	-	-
miR-186-5p	1.27	-	-	0.82	-	0.83	-	-	-	-
miR-1260b	1.23	-	-	0.76	-	0.79	-	-0.71	-	-
miR-6085	1.17	-	-	0.72	-	0.77	-	-0.81	-	-
miR-142-3p	0.99	-	-	0.80	-	0.77	-0.53	-	-	-
miR-29b-3p	0.97	-	-	-	-	-	-0.66	-	-	-
miR-29c-3p	0.94	-	-	-	-	-	-0.60	-	-	-
miR-155-5p	0.90	0.67	-	0.77	-	0.81	-	-	-0.59	-0.58
miR-874-3p	0.89	-	-	0.64	-	0.67	-	-	-	-
miR-30c-5p	0.81	-	-	0.81	-	0.82	-	-0.77	-	-
miR-5100	0.79	-	0.64	0.76	-	0.79	-	-0.69	-	-
miR-146b-5p	0.79	-	-	0.92	-	0.89	-	-	-	-
miR-7977	0.70	-	-	-	-	-	-	-0.73	-	-
miR-664b-3p	0.68	-	-	0.75	0.65	0.76	-	-	-	-
miR-30b-5p	0.63	-	-	0.75	-	0.72	-	-	-	-
miR-199a-3p	-0.64	-	-	-	-	-	-	-	0.59	0.56

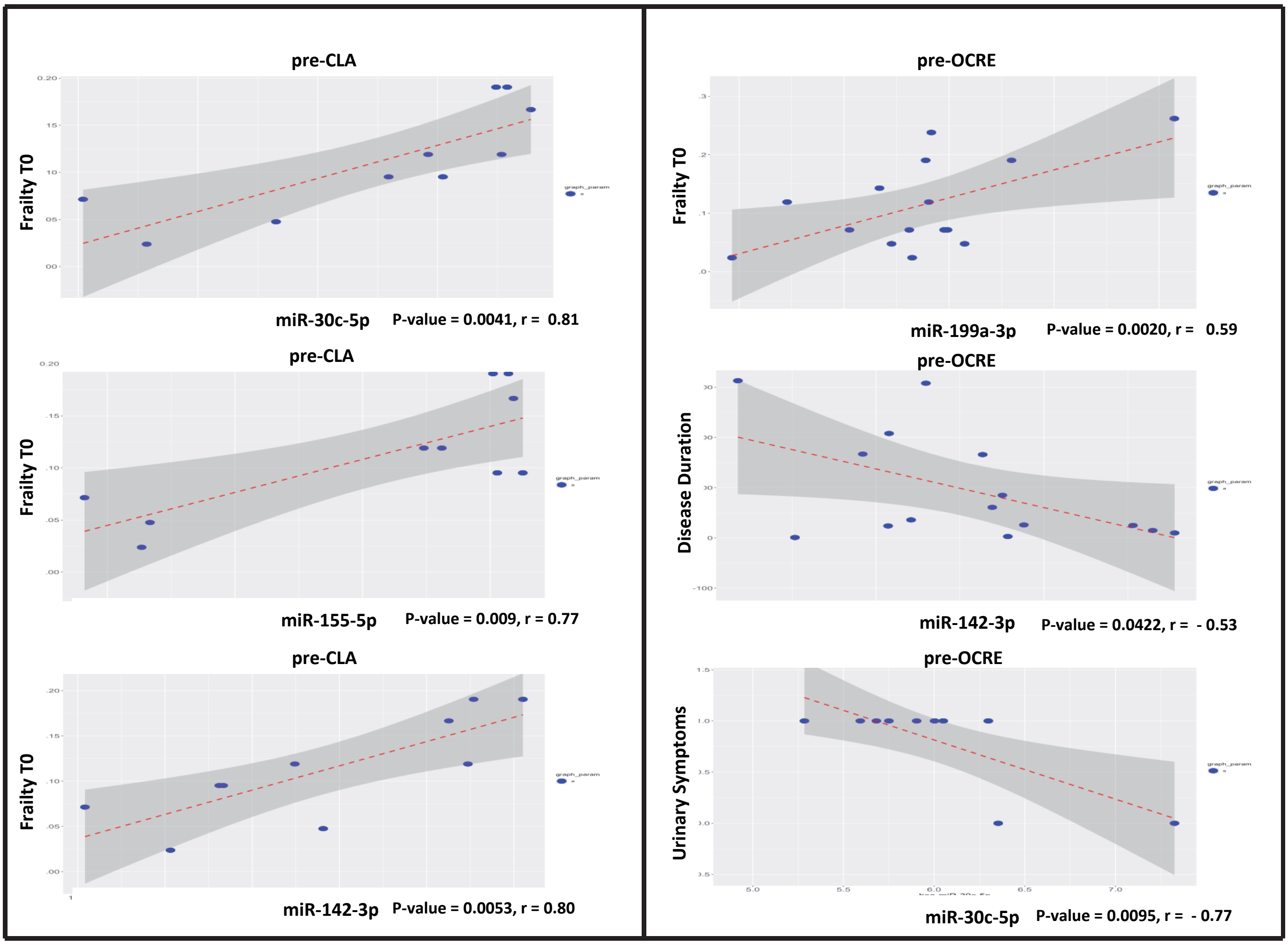

Supplementary Figure S4

		CLA T1			OCRE T1		
	**Log2FC (CLA T1 vs OCRE T1)**	**Urinary Symptoms**	**FI T0**	**FI T1**	**Urinary Symptoms**	**FI T0**	**FI T1**
**miR-1202**	-0.68	-	-	-	-	-	-
**miR-1268b**	-0.79	-	-	-	-	-	-
**miR-4442**	-0.76	-	-	-	-	-	-
**miR-4443**	-0.85	-	-	-	-	-	-
**miR-4507**	-0.86	-	-	-	-	-	-
**miR-4530**	-0.90	-	-	-	-	-	-
**miR-4713-3p**	-0.71	-	-0.63	-0.64	0.69	-	-
**miR-6131**	-0.61	-	-	-	0.74	-	-
**miR-6165**	-0.63	-	-	-	-	-	-
**miR-6740-5p**	-0.81	-	-	-	-	-	-
**miR-6763-5p**	-0.83	-	-	-	-	-	-
**miR-6780b-5p**	-0.83	-	-	-0.64	-	-	-

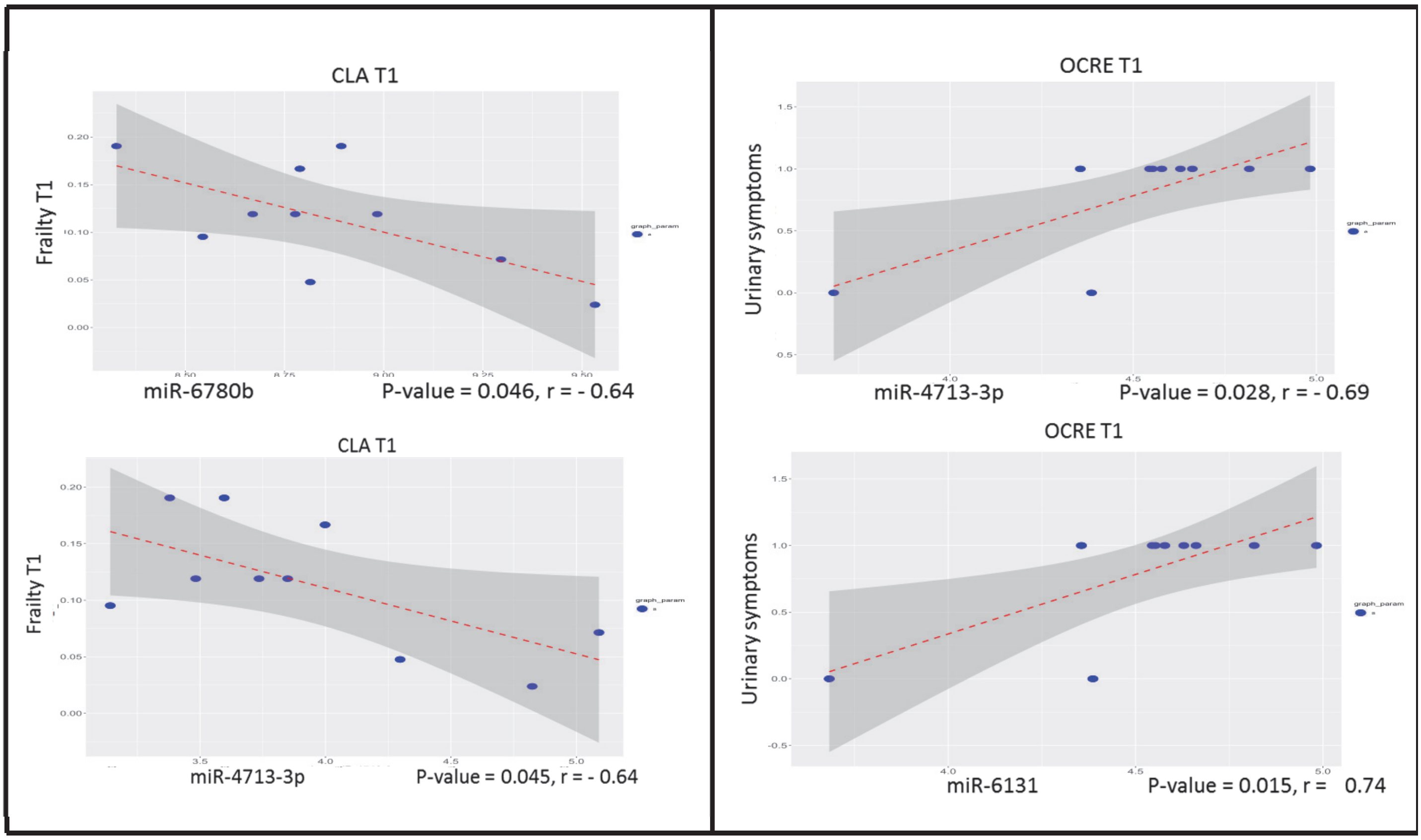

Supplementary Figure S5

		CLA T0		CLA T1			
	log_2FC (CLA T1 VS PRE-CLA)	Urinary symptoms	FI T1	Disease Duration at T0	Urinary symptoms	FI T0	FI T1
miR-584-5p	0.86	0.66	-	-	-	-	-
miR-199a-3p	0.75	-	-	-	-0.71	-	-
miR-151a-3p	0.74	-	-	-	-0.75	-0.67	-0.68
miR-326	0.73	-	-	0.74	-	-	-
miR-221-3p	0.70	0.68	-	0.83	-	-	-
miR-151a-5p	0.67	0.71	-	-	-0.75	-	-
miR-23b-3p	0.66	0.74	-	0.88	-	-	-
miR-148b-3p	0.62	0.75	-	-	-	-	-
miR-27b-3p	0.61	-	-	0.76	-	-	-
miR-342-5p	-0.59	-	-	-0.80	-	-	-
miR-8069	-0.60	-	-	-	0.81	-	-
miR-4443	-0.61	-0.79	-	-	-	-	-
miR-4507	-0.62	-0.83	-	-	-	-	-
miR-150-5p	-0.63	-	-	-0.74	-	-	-
miR-29c-3p	-0.63	-	-	-	0.88	-	-
miR-4505	-0.64	-0.72	-	-	-	-	-
miR-197-3p	-0.67	-	0.66	-	-	-	-

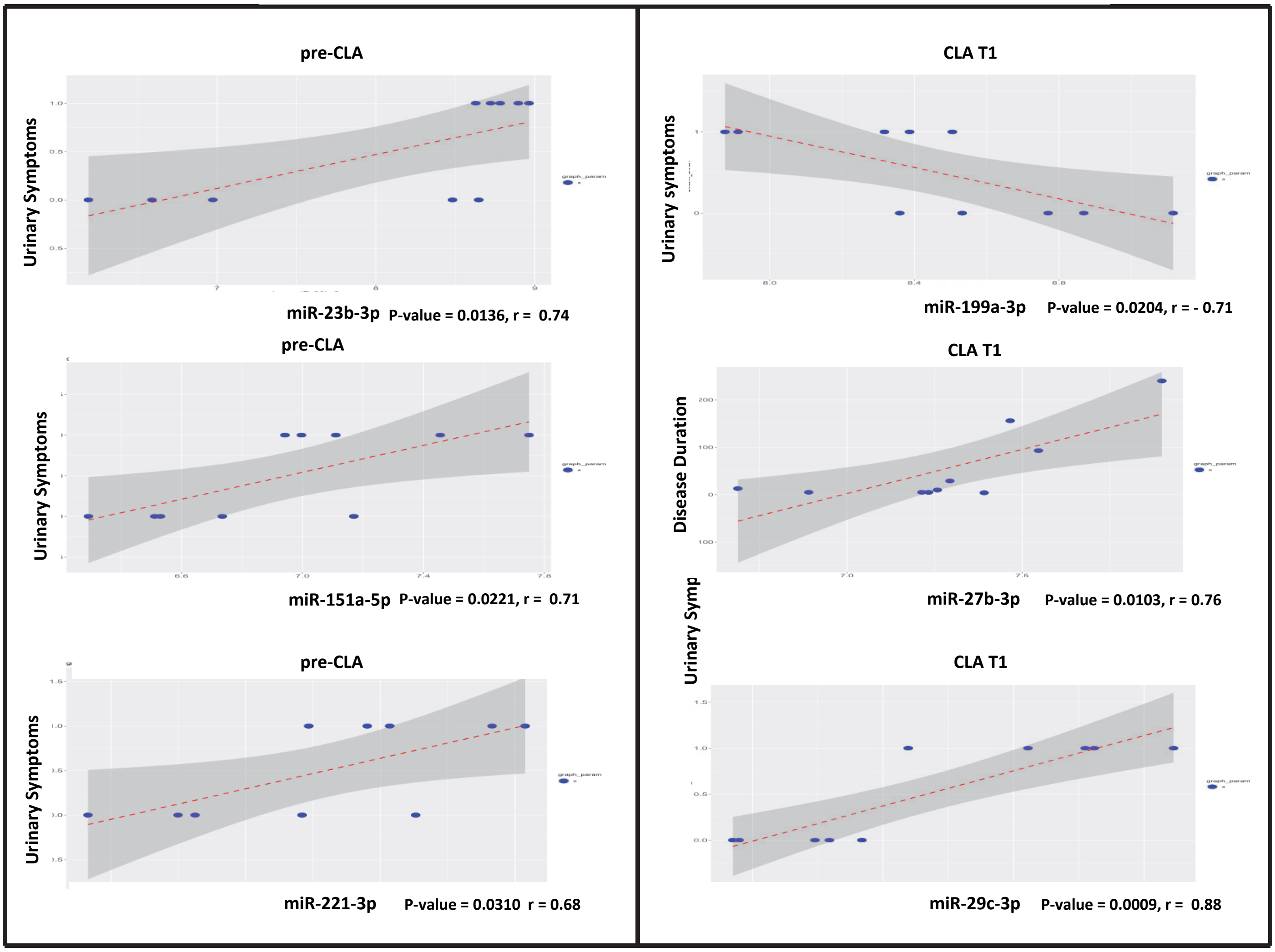

Supplementary Figure S6

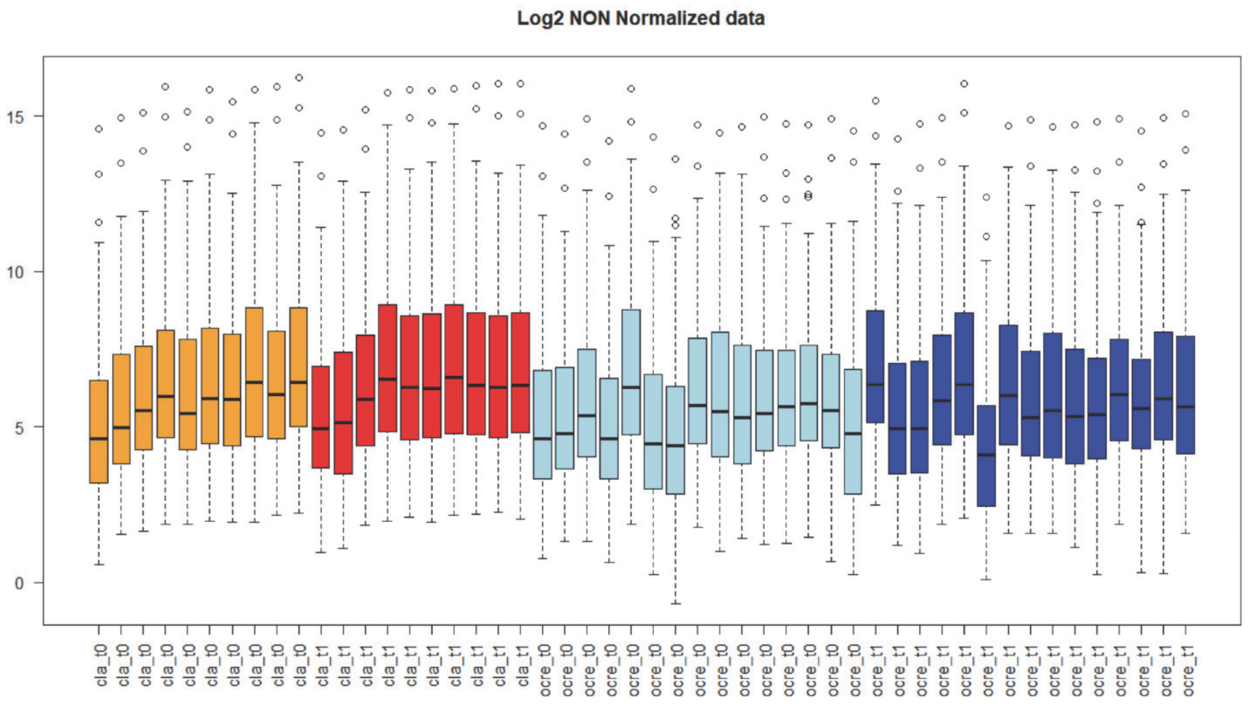

Log2 data normalized (aligned) to median

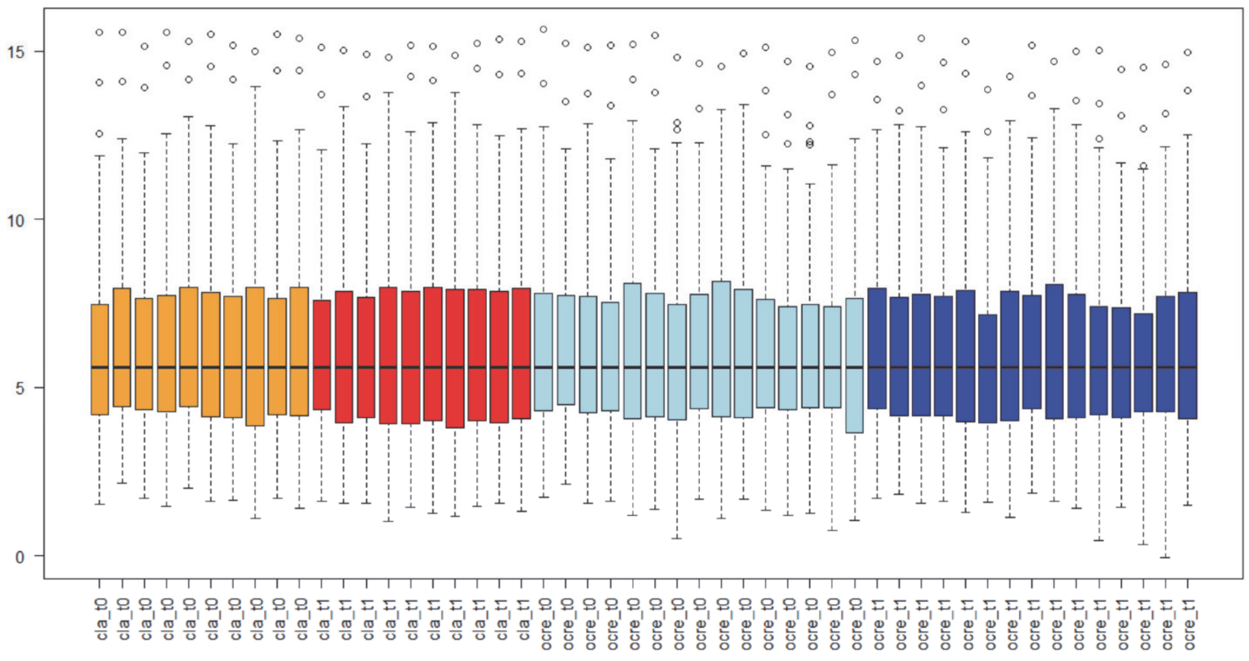

Supplementary Figure S7

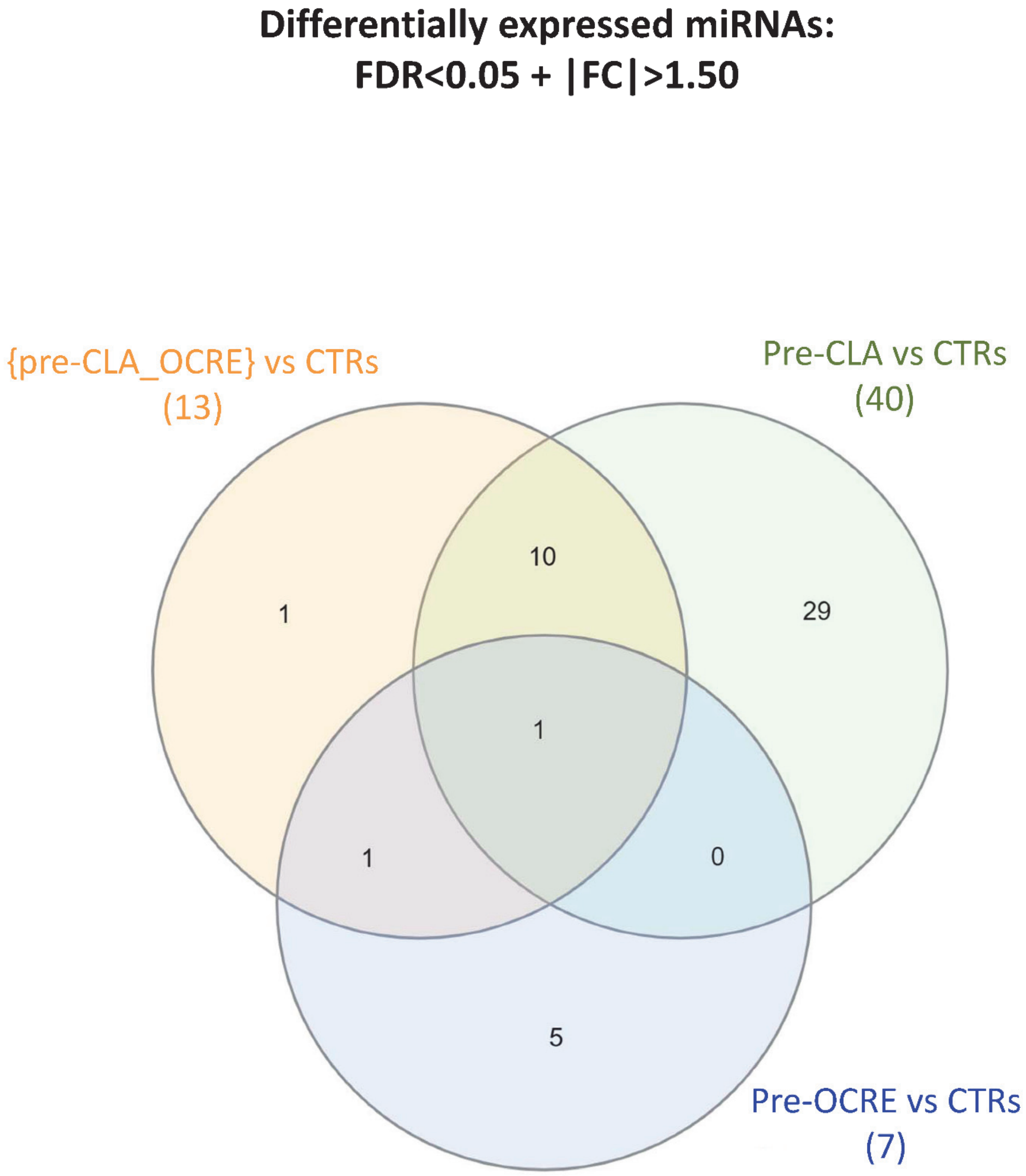

Supplementary Figure S8

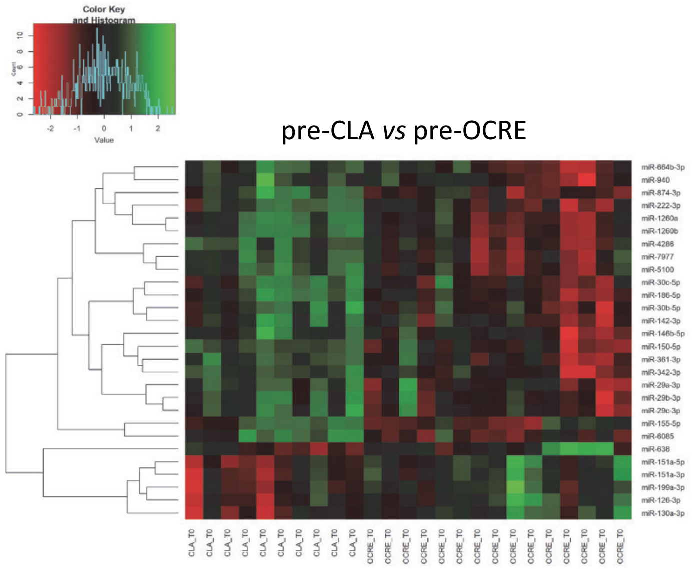

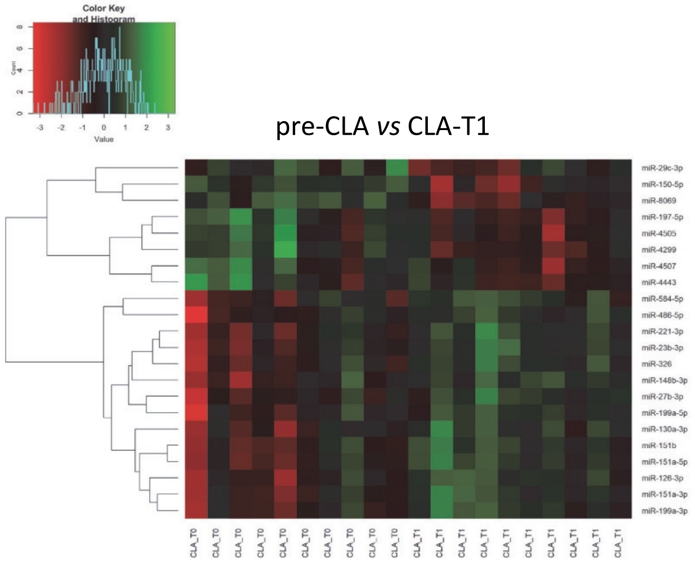

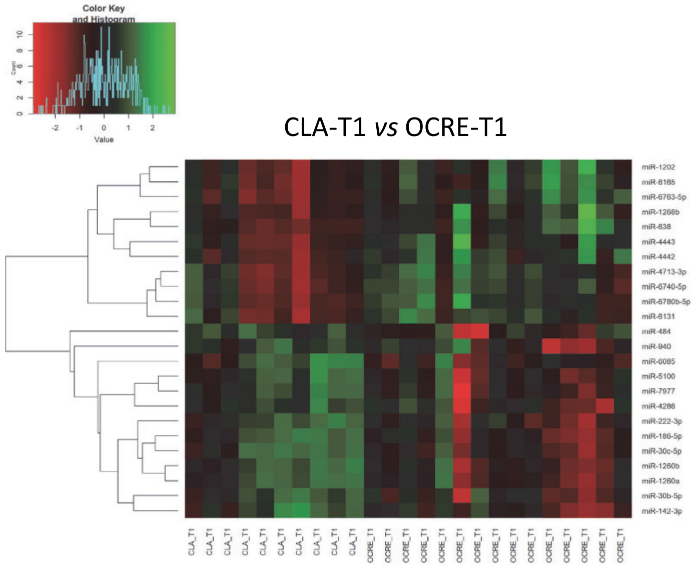

Supplement: Supplementary file 1 [file DataSheet_1.pdf]
