# Supplementary material for: Cladribine and ocrelizumab induce differential miRNA profiles in peripheral blood mononucleated cells from relapsing–remitting multiple sclerosis patients
Source: Front Immunol. 2023 Dec 13;14:1234869. doi: 10.3389/fimmu.2023.1234869 (PMC10751352; doi:10.3389/fimmu.2023.1234869)
Supplement: Supplementary file 2 [file DataSheet_2.pdf]

Supplementary Material

Cladribine and Ocrelizumab induce differential miRNA profiles in peripheral blood mononuclear cells from relapsing-remitting Multiple Sclerosis patients.

Ivan Arisi[1,2*], Leonardo Malimpensa[3*], Valeria Manzini[1], Rossella Brandi[1], Tommaso Gosetti di Sturmeck[1], Chiara D'Amelio[1], Sebastiano Crisafulli[4], Gina Ferrazzano[5], Daniele Belvisi[3,5], Francesca Malerba[1], Rita Florio[1], Esterina Pascale[6], Hermona Soreq[7], Marco Salvetti[3,8], Antonino Cattaneo[1,9], Mara D'Onofrio[1†], Antonella Conte[3,5†]

[1] European Brain Research Institute (EBRI) Rita Levi-Montalcini, Rome, Italy,

[2] Institute of Translational Pharmacology, National Research Council, Rome, Italy,

[3] IRCCS Istituto Neurologico Mediterraneo Neuromed, Pozzilli (IS), Italy,

[4] Fondazione IRCCS Istituto Neurologico Carlo Besta, Milan, Italy,

[5] Department of Human Neurosciences, Sapienza University of Rome, Rome, Italy,

[6] Department of Medical-Surgical Sciences and of Biotechnologies, "Sapienza" University of Rome, Rome, Italy

[7] The Edmond and Lily Safra Center of Brain Science and The Life Sciences Institute, The Hebrew University of Jersusalem, Jerusalem, Israel,

[8] Centre for Experimental Neurological Therapies (CENTERS), Department of Neurosciences, Mental Health and Sensory Organs, Sapienza University of Rome, Italy,

[9] Bio@SNS Laboratory of Biology, Scuola Normale Superiore, Pisa, Italy.

* Frist authorship

† Senior authorship

*** Correspondence:**

Prof. Antonella Conte

Department of Human Neurosciences, “Sapienza" University

P. le A. Moro 5

Rome, Italy, 00185

antonella.conte@uniroma1.it

Supplementary Table S1: miRNA profiles on PBMC of RRMS patients before either CLA or OCRE treatment (T0 in pre-CLA vs pre-OCRE) compared to known literature.

Supplementary Table S2: miRNA profiles on PBMCs post-treatment, T1 (CLA T1 vs OCRE T1) compared to known literature.

Supplementary Figure S1. miRNA profile levels on PBMC in post-treatment groups (T1).
1A) Venn Diagram for the DE miRNAs in pretreatments and post treatments groups, for CLA and OCRE. **1B)** Volcano plot of the CLA T1 vs OCRE T1 miRNAs comparison. DE miRNAs (down-regulated in green and up-regulated in red) were selected by the R limma tool with thresholds FDR<0.05 and linear FoldChange >1.5. **1C)** DE miRNAs in CLA T1 vs OCRE T1 comparison. Log2 median normalized expression values of DE miRNAs are shown; miRNAs were selected using the following thresholds: R limma test FDR<0.05 + linear FoldChange >1.5. Boxes correspond to median ± IQR, black dots are outliers.

Supplementary Figure S2: OCRE-T1 vs pre-OCRE effect on miRNA DE profiles. 2A) Volcano plot of the OCRE T1 vs pre-OCRE microRNAs comparison. **2B**) Multidimensional Scaling of CLA samples based on the miRNA transcriptome. Each arrow corresponds to a different patient treated with OCRE and connects the miRNAs profile at T0 (light blue dot) to the profile at T1 (dark blue dot).

Supplementary Table S3: Effects of CLA treatment on PBMCs miRNA profiles at T1. Data comparison to known literature.

Supplementary Table S4: miRNA profile values in different group comparison. Different comparisons between groups present DE miRNAs which are significantly overexpressed (red), downregulated (green) or non-significantly deregulated (black). Log2 median normalized expression values of DE miRNAs are shown; miRNAs were selected using the following thresholds: R limma test FDR<0.05 + linear FoldChange >1.5.

Supplementary Table S5: Statistical comparisons between CLA and OCRE treated patients for demographics and clinical data. Either two-sided Mann-Witney or Fisher's exact test p-values are shown where appropriate.

Supplementary Table S6: Pearson's correlation between miRNAs expression values and lymphocytes counts. Pre-CLA and pre-OCRE groups are correlated to counts at T0, while CLA T1 and OCRE T1 groups are correlated to counts at T1.

tatistical comparisons between CLA and OCRE treated patients for demographics and clinical data. Either two-sided Mann-Witney or Fisher's exact test p-values are shown where appropriate.

Supplementary Figure S3: Correlation between miRNA profiles and clinical data in pre-treatment groups. 3A) The Pearson correlation index between DE miRNA (selected by the pre-CLA vs pre-OCRE contrast) and specific clinical variables is shown in case of statistical significance. T1 = 6 months after treatment start. Analyzed clinical variables: presence of urinary symptoms (yes/no, binary); EDSS score (integer discrete); Frailty Index score; Disease duration in months**.** Table of

significant correlations**. 3B)** Scatter plot expression value vs clinical variable for selected correlations with miRNAs in the different pre-CLA and pre-OCRE groups; the regression line is drawn.

Supplementary Figure S4: Correlation between miRNA profiles and clinical data in post-treatment groups. 4A) The Pearson correlation index between DE miRNA (CLA T1 vs OCRE T1) and selected clinical variables is shown in case of statistical significance. T1 = 6 months after treatment starts. Analyzed clinical variables: presence of urinary symptoms (yes/no, binary); EDSS score (integer discrete); Frailty Index score; Disease duration in months**.** Table of significant correlations. **4B)** Scatter plot expression value vs clinical variable for selected correlations with miRNAs in the OCRE T1 and CLA T1 groups; the regression line is drawn. Scatter plot expression value vs clinical variable for selected correlations with miRNA in both groups; the regression line is drawn

Supplementary Figure S5: Correlation between miRNA profiles and clinical data in pre and post CLA treatment. 5A) The Pearson correlation index between DE miRNA (pre- CLA vs CLA T1) and selected clinical variables is shown in case of statistical significance. T1 = 6 months after treatment start. Analyzed clinical variables: presence of urinary symptoms (yes/no, binary); EDSS score (integer discrete); Frailty Index score; Disease duration in months**.** Table of significant correlations. 5**B)** Scatter plot expression value vs clinical variable for selected correlations with miRNA in the pre-CLA and CLA T1 groups; the regression line is drawn.

Supplementary Figure S6: Boxplots non-normalized and median normalized of Log2 transformed data samples.

Supplementary Figure S7: Venn diagram with numbers of DEGs, *vs* controls, for pooled pre-CLA and pre-OCRE groups, compared to pre-CLA and pre-OCRE groups separately analysed. DE miRNAs were selected by the R limma tool with thresholds FDR<0.05 and linear FoldChange >1.5.

Supplementary Figure S8: Heat maps of DEGs in different comparisons. The normalised expression value for each miRNA is mean-centered.

Supplementary Table S1.

miRNA profiles on PBMC of RRMS patients before either CLA or OCRE treatment (T0 in pre-CLA vs pre-OCRE) compared to known literature		
**miRNAs**	**Log_2FC**	**Previous studies on MS and other neurodegenerative diseases**
**miR-1260a**	**1.60**	
**miR-186-5p**	**1.42**	↑ in MS *(Angerstein et al 2012)*, dysregulated in MS *(Keller et al., 2013)*, ↑ decreased CXCL13 expression, alleviating neuropathic pain *(Jiang et al., 2016)*
**miR-1260b**	**1.30**	↑ in Addison's Disease *(Fichna et al., 2021)*, ↓ in glaucoma *(Martinez et al., 2021)*
**miR-6085**	**1.27**	
**miR-142-3p**	**1.09**	↑ in MS with ↓ IL-1β *(De Vito et al., 2021, Mandolesi et al., 2017),* ↑ in MS *(Arruda et al., 2014, Ma et al., 2014, Luo et al., 2018)*
**miR-29b-3p**	**1.01**	↑ in RRMS in $CD4^+T$, ↓ after INF-β *(Fattahi et al., 2018)*, ↓ Th_{17} e IL-17A after IFN-β treatment, whereas miRNA levels remained constant *(Karimi et al., 2021)*
**miR-29c-3p**	**0.96**	↓ in MS *(Gandhi et al., 2015)*, ↓ in GMLS (gray matter lesions in MS) (*Tripathi et al., 2021)* ↓ in MS *(Hecker et* al., 2013)
**miR-30c-5p**	**0.92**	↑ in MS (both RR and PP) in CSF *(Amoruso et al., 2020)*, ↓ in CI (cognitive impairment) vs non-CI MS patients *(Lovera et al., 2020)*, ↓ PD *(Yourefi et al., 2022)*, ↓ in sALS *(Liguori et al., 2018)*
**miR-150-5p**	**0.92**	↑ in MS and remains ↑ after Natalizumab treatment *(Bergman et al., 2016)*, miRNA enriched in microglia controlling synaptic plasticity and cognition *(Scaroni et al, 2022)*
**miR-874-3p**	**0.90**	↑ in PD *(Chen et al., 2020)*, ↑ in hippocampal sclerosis *(Bencurova et al., 2017)*, ↓ in AD *(Van den Berg et al., 2019)*
**miR-155-5p**	**0.88**	↑ in MS *(Maciak et al., 2021)*, ↑ in MS *(McCoy et al., 2017)*, ↓ in SPMS targeting SOCS6 in $CD4^+T$ *(Sanders et al., 2016)*
**miR-146b-5p**	**0.86**	↑ in MS, ↓ after dmf treatment which targets NF-kβ pathway to avoid Th transition *(Giuliani et al., 2021)*, ↑ in MS correlated with EDSS *(Mozo et al., 2022)*, ↑ in MS *(Magheri et al., 2022)*
**miR-940**	**0.86**	possible biomarker for AD *(Zakeri et al., 2020)*
**miR-5100**	**0.80**	↑ in familial MS different from sporadic MS *(Altun et al., 2017, Harirchian et al., 2018)*, ↑ in AD *(Sun et al., 2021)*
**miR-29a-3p**	**0.78**	↓ in MS *(Chen et al., 2018),* ↓ in MS *(Ghandi et al., 2015)* ↓ In RRMS *(Dolati et al., 2018)*
**miR-222-3p**	**0.73**	↑ in TSC *(Trelinska et al., 2016)* ↑ in Huntington Disease *(Phanelles et al., 2016)*, ↓ in PD *(Chen et al., 2018)*

**miR-30b-5p**	**0.71**	↓ in RRMS *(Groen et al., 2018)*, biomarker for RRMS, AD and PD *(Ebrahimkhani et al., 2017)*, ↓ in NMOSD (Neuromyelitis Optica Spectrum disorders) *(Chen et al., 2017)*, dysregulated in all neurodegenerative diseases *(Brennan et al., 2019)*
**miR-7977**	**0.70**	↑ in ALS *(Chen et al., 2021)* ↑ in $CD4^{+}$T in Addison's Disease *(Fichna et al., 2021)*
**miR-4286**	**0.68**	↑ in MS, targets synaptotagmin-7 (syt7) *(Fritsche et al., 2019)*
**miR-664b-3p**	**0.67**	↑ in AD *(Wen et al., 2022)*
**miR-342-3p**	**0.64**	↑ in RRMS *(Dolati et al., 2018)*, ↑ in ALS *(Wen-Lo et al., 2021)* ↓ in AD *(Chen et al., 2018)*
**miR-361-3p**	**0.60**	↑ in AD inhibits β-amyloid *(Ji et al., 2019)* in mice, ↓ in Huntington disease *(Dhawan 2021)*
**miR-638**	**-0.91**	↓ in MS *(Mandegarfard et al., 2022)* ↓ in the skeletal muscle in ALS patients *(Menges et al., 2021)*
**miR-130a-3p**	**-0.79**	↑ in MS *(Hanselmann et al., 2020)*, ↑ in TSC *(Trelinska et al., 2016)*, ↓ in ALS *(Liguori et al., 2018)*, ↓ in AD *(Lu et al., 2022)* ↓ in Chron's Disease it provokes ↑ inflammation *(Pang et al., 2020)*, ↓ in psoriasis *(Huang et al., 2022, Alatas et al., 2020)*
**miR-151a-5p**	**-0.69**	↓ in MS (white matter lesions) *(Hanselmann et al., 2020)*, ↑ ALS *(Dobrowolny et al., 2021)* ↑ in PD *(Tong et al., 2022)*
**miR-126-3p**	**-0.68**	↓ in MS after Natalizumab treatment *(Mancuso et al., 2022, Meira et al., 2014)*
**miR-151a-3p**	**-0.68**	↓ in MS *(Keller et al., 2013)*, ↑ in ischemic stroke *(Sorensen et al., 2014)*, ↑autism spectrum disorder (*Asfar et al., 2019)*
**miR-199a-3p**	**-0.64**	↓ serum MS vs HC ↓ remitting vs relapsing *(Regev et al., 2018)*, it promotes ERK-mediated IL-10 production in systemic lupus erythematosus *(Su et al., 2019)*

Supplementary Table S2

**miRNA profiles on PBMCs post-treatment, T1 (CLA T1 vs OCRE T1) compared to known literature**		
**miRNAs**	**Log_2FC**	**Previous studies on MS and other neurodegenerative diseases**
**miR-4530**	**-0.90**	↓in ALS (Taguchi et al., 2018), dysregulated in ALS *(Kmetzsch et al.,2022)*
**miR-4507**	**-0.86**	↓ in ALS *(Katsu et al., 2019)*
**miR-4443**	**-0.85**	↑ in PD (Salemi et al., 2021), dysregulated in ALS *(Wen Lo et al.,* 2021), It induces $CD4^+$ dysfunction *(Qi et al, 2017; Li et al, 2020)*
**miR-6780b-5p**	**-0.83**	
**miR-6763-5p**	**-0.83**	
**miR-6740-5p**	**-0.81**	↑ in Ossessive-Compulsive Disorder *(Aydın et al., 2022)*
**miR-1268b**	**-0.79**	↑ in psoriasis *(Solvin et al., 2021)*, biomarker of ischemic stroke *(Deng et al., 2022)*
**miR-4442**	**-0.76**	↓ in idiopathic inflammatory myopathy (IIM) *(Chen et al., 2018)*
**miR-4713-3p**	**-0.71**	
**miR-1202**	**-0.68**	↑ in hippocampal sclerosis *(Gattas et al., 2022),* ↑ glutamate receptor-4 (GRM4) in major depression state (MD) *(Wang et al., 2021, Chakraborty et al., 2019)*
**miR-6165**	**-0.63**	biomarker of Moyamoya Disease (MMD) *(Wang et al., 2021),* ↑ in apoptotic role provoked by p75NTR *(Shi et al., 2015)*
**miR-6131**	**-0.61**	↑ in AD *(Lu et al., 2021)*

Supplementary Table S3

Effects of CLA treatment on PBMCs miRNA profiles at T1. Data comparison to known literature		
miRNAs	Log_2FC	**Previous studies on MS and other neurodegenerative diseases**
**miR-151a-3p**	**0.76**	↓ in MS *(Keller et al., 2013),* ↑ in ischemic stroke *(Sorensen et al., 2014)*, ↑Autism
**miR-130a-3p**	**0.78**	↑ in MS *(Hanselmann et al., 2020),* ↑ in TSC *(Trelinska et al., 2016*), ↓ in ALS *(Liguori et al., 2018)*, ↓ in AD *(Lu et al., 2022)* ↓ in Chron's Disease it provokes ↑ inflammation *(Pang et al., 2020),* ↓ in psoriasis *(Huang et al., 2022, Alatas et al., 2020)*
**miR-126-3p**	**0.81**	↓ in MS after Natalizumab treatment *(Mancuso et al., 2022, Meira et al., 2014)*
**miR-151a-5p**	**0.63**	↓ in MS (white matter lesions) *(Hanselmann et al., 2020),* ↑ ALS *(Dobrowolny et al., 2021)* ↑ in PD *(Tong et al., 2022)*
**miR-151b**	**0.60**	↓ in sALS *(Liguori et al., 2018)*
**miR-326**	**0.85**	↑ MS active lesions ↓ after Natalizumab treatment *(Gallardo Perez et al., 2022)*, ↑ in RRMS, involved in Th17 maturation and differentiation *(Azimi et al., 2019*), ↑ inflammation (IFN-γ, IL-17) *(Fattahi et al., 2019, Karimi et al., 2020)*
**miR-584-5p**	**0.96**	dysregulated in MS *(Angersten et al., 2012),* dysregulated in pediatric Epilepsy (Gu et al., 2020), ↑ in IV lupus nephritis *(Quiroz et al., 2017)*
**miR-199a-3p**	**0.66**	↓ serum MS vs HC ↓ remitting vs relapsing *(Regev et al., 2018)* it promotes ERK-mediated IL-10 production in systemic lupus erythematosus *(Su et al., 2019)*
**miR-23b-3p**	**0.64**	↑ in remitting MS *(Baulina et al., 2018)*, ↑ keeps Th/Treg balanced in intracranial aneurysm *(Sun et al., 2020)*
**miR-221-3p**	**0.69**	↑ in MS and targets immunological, autophagic and ATPases components *(Liguori et al., 2017),* biomarker in PD *(Schultz et al., 2019)*
**miR-27b-3p**	**0.60**	↑ in MS *(Dolati et al., 2018),* ↑ in MS *(Hanselmann et al., 2022)* ↓ in MS, it generally keeps the BBB less permeable targeting Clandin-5 in the WNT/β-catenin pathway *(Harati et al., 2022)*
**miR-199a-5p**	**0.77**	↓ in remitting phase in CD+T cells *(Ghadiri et al., 2018),* ↓ in MS *(Luo et al., 2018)*
**miR-486-5p**	**0.76**	↓ in MS (Manna et al., 2018), dysregulated in MS *(Martinez et al., 2022, Regev et al., 2018)*
**miR-148b-3p**	**0.60**	↓ in MS *(Liguori et al., 2018),* it is a MS biomarker targeting ECMR, PI3K-Akt, TGFβ *(Prabahar etal., 2022)*
**miR-150-5p**	**-0.62**	↑ in MS and remains ↑ after Natalizumab treatment *(Bergman et al., 2016),* miR enriched in microglia controlling synaptic plasticity and cognition *(Scaroni et al, 2022)*
**miR-197-5p**	**-0.63**	↓ in MS in T-cells and targets TNFSF14 *(Jernas et al., 2013),* dysregulated in Autism *(Kichukova et al., 2021),* ↑ in TLE *(Yakovleva et al., 2022)*

**miR-29c-3p**	**-0.63**	↓ in MS *(Gandhi et al., 2015)*, ↓ in GMLS (gray matter lesions in MS*) (Tripathi et al., 2021)* ↓ in MS *(Hecker et al., 2013)*
**miR-342-5p**	**-0.58**	↑ in RRMS *(Ma et al., 2014)*, ↑ in MS *(Gandhi et al., 2015, Hecker et al., 2013)*, It regulates Neural Stem Cell Differentiation *(Gao et al, 2017).* It is required for TNFα microglia activation *(Bras et al, 2020)*
**miR-4443**	**-0.60**	↑ in PD *(Salemi et al., 2021)*, dysregulated in ALS *(Wen Lo et al., 2021)*, It induces CD4+ dysfunction *(Qi et al, 2017; Li et al, 2020)*
**miR-4505**	**-0.64**	↑ in ALS *(Freischmidt et al., 2014)*
**miR-4507**	**-0.61**	↓ in ALS (Katsu et al., 2019)
**miR- 8069**	**-0.59**	

Supplementary Table S4

CLA T1 vs pre-CLA		pre-CLA vs HC	CLA T1 vs HC	pre-CLA vs pre-OCRE
**miRNAs**	**Log$_2$FC**	**Log$_2$FC**	**Log$_2$FC**	**Log$_2$FC**
miR-584-5p	**0.856**	**-0.953**	-0.053	**-0.544**
miR-130a-3p	**0.817**	**-1.090**	-0.232	**-0.795**
miR-126-3p	**0.802**	**-1.151**	-0.330	**-0.678**
miR-199a-5p	**0.783**	**-0.838**	-0.057	-0.187
miR-486-5p	**0.748**	**-0.777**	-0.006	**-0.688**
miR-199a-3p	**0.746**	**-1.135**	-0.383	**-0.642**
miR-151a-3p	**0.740**	**-1.192**	-0.434	**-0.676**
miR-326	**0.726**	-0.221	**0.537**	-0.238
miR-221-3p	**0.700**	**-0.714**	0.001	-0.332
miR-151a-5p	**0.668**	**-0.906**	-0.218	**-0.694**
miR-23b-3p	**0.656**	**-0.638**	0.036	**-0.506**
miR-148b-3p	**0.615**	-0.244	0.365	-0.215
miR-27b-3p	**0.608**	**-0.770**	-0.161	-0.420
miR-151b	**0.603**	**-0.681**	-0.068	**-0.554**
miR-652-3p	**0.536**	-0.366	0.171	-0.400
miR-22-3p	**0.503**	**-0.727**	-0.224	-0.468
miR-324-5p	0.495	-0.413	0.083	-0.478
miR-185-5p	0.468	**-0.612**	-0.144	-0.313
miR-24-3p	0.335	-0.368	-0.033	-0.180
miR-17-5p	0.307	-0.391	-0.084	-0.125
miR-376c-3p	0.258	**-1.401**	**-1.144**	**-0.680**
miR-376a-3p	0.208	**-1.181**	**-0.973**	-0.403
miR-197-3p	-0.022	**0.648**	**0.625**	0.227
let-7c-5p	-0.033	0.466	0.433	0.195
miR-181b-5p	-0.153	**0.619**	0.466	0.254
miR-186-5p	-0.203	**0.830**	**0.628**	**1.416**
miR-6826-5p	-0.209	-0.393	**-0.602**	-0.381
miR-5100	-0.292	**0.950**	**0.658**	**0.799**
miR-6165	-0.304	**-0.571**	**-0.876**	-0.372
miR-1268b	-0.307	**-0.773**	**-1.079**	-0.441
miR-1260a	-0.326	**1.628**	**1.302**	**1.599**
miR-1202	-0.335	**-0.634**	**-0.968**	-0.375
miR-4281	-0.341	-0.386	**-0.728**	-0.081
miR-1260b	-0.360	**1.232**	**0.871**	**1.303**
miR-6763-5p	-0.361	**-0.522**	**-0.883**	-0.416
miR-1207-5p	-0.371	-0.390	**-0.761**	-0.130
miR-874-3p	-0.382	**0.684**	0.303	**0.895**
miR-940	-0.422	**1.290**	**0.867**	**0.856**
miR-3162-5p	-0.429	-0.247	**-0.676**	-0.124
miR-6740-5p	-0.476	**-0.618**	**-1.093**	-0.495
miR-6717-5p	-0.477	**-0.574**	**-1.051**	-0.337
miR-3198	-0.479	**-0.742**	**-1.221**	-0.293
miR-4713-3p	-0.479	-0.291	**-0.771**	-0.349
miR-6879-5p	-0.486	-0.138	**-0.625**	-0.209
miR-6127	-0.495	-0.325	**-0.820**	-0.137
miR-6131	**-0.504**	-0.425	**-0.929**	-0.271
miR-4442	**-0.514**	-0.375	**-0.889**	-0.208
miR-638	**-0.539**	**-0.856**	**-1.395**	**-0.908**
miR-29b-3p	**-0.568**	**0.743**	0.175	**1.009**
miR-6780b-5p	**-0.579**	-0.321	**-0.901**	-0.330
miR-342-5p	**-0.586**	0.319	-0.256	0.422
miR-8069	**-0.598**	0.287	-0.306	**0.562**
miR-4443	**-0.609**	0.124	-0.468	-0.292
miR-4507	**-0.618**	0.004	**-0.591**	-0.079
miR-150-5p	**-0.626**	**0.525**	-0.111	**0.920**
miR-29c-3p	**-0.630**	**0.549**	-0.089	**0.962**
miR-4505	**-0.641**	0.103	**-0.523**	0.257
miR-197-5p	**-0.667**	-0.027	**-0.712**	0.212

Supplementary Table S5

	Age	Sex	Disease Duration at T1 (years)	EDSS T0	EDSS T1	FI T0	FI T1	Previous Therapy	New lesions T0	GD+ Enhacing Lesions T0	n. of relapses in the last 6 month
**CLA: Mean +- SD**	39.0 ± 7.5		5.6 ± 6.9	1.5 ± 1.1	1.5 ± 1.2	0.11 ± 0.06	0.11 ± 0.06				
**OCRE: Mean +- SD**	40.5 ± 10.4		8.8 ± 9.1	2.6 ± 1.8	2.2 ± 1.8	0.11 ± 0.07	0.11 ± 0.07				
**M-W test, two-sided, CLA vs OCRE**	0.757		0.194	0.052	0.407	0.912	0.889				
**Fisher's test, two-sided, CLA vs OCRE**		1.000						0.241	1.000	0.241	1.000

Supplementary Table S6

	pre-CLA			CLA T1			pre-OCRE			OCRE T1		
	White_cells	Neutrophils	Lymphocytes	White_cells	Neutrophils	Lymphocytes	White_cells	Neutrophils	Lymphocytes	White_cells	Neutrophils	Lymphocytes
hsa-let-7a-5p	0.676	0.572	0.528	0.295	0.373	0.028	0.380	0.409	0.147	0.408	0.139	0.263
hsa-let-7b-5p	0.517	0.472	0.192	0.051	0.257	-0.310	-0.078	0.105	-0.272	0.260	0.174	0.072
hsa-let-7c-5p	0.035	-0.171	0.565	-0.402	-0.397	-0.030	0.194	0.436	-0.399	0.364	0.325	0.021
hsa-let-7d-5p	0.214	0.062	0.485	0.116	0.280	-0.095	0.381	0.454	0.050	0.387	0.267	0.089
hsa-let-7e-5p	0.314	0.283	0.059	0.233	0.488	-0.303	0.012	0.217	-0.300	0.006	0.171	-0.226
hsa-let-7f-5p	0.307	0.277	0.311	0.318	0.029	0.647	0.543	0.469	0.355	0.456	0.170	0.281
hsa-let-7g-5p	0.342	0.381	0.148	0.027	-0.207	0.444	0.493	0.346	0.544	0.391	-0.119	0.537
hsa-let-7i-5p	0.727	0.747	0.196	0.427	0.511	0.055	0.409	0.268	0.580	0.403	0.090	0.297
hsa-miR-101-3p	-0.220	-0.145	-0.107	-0.285	-0.459	0.195	0.469	0.323	0.395	0.385	0.129	0.273
hsa-miR-103a-3p	0.355	0.318	0.100	0.265	0.530	-0.297	0.203	0.304	-0.094	0.355	0.320	-0.014
hsa-miR-106b-5p	0.085	0.199	-0.201	-0.207	-0.267	0.075	0.482	0.288	0.505	0.329	0.071	0.250
hsa-miR-107	0.120	-0.049	0.390	0.236	0.327	0.105	0.325	0.377	0.066	0.380	0.338	-0.003
hsa-miR-1202	0.556	0.557	0.042	0.224	0.265	0.001	-0.023	-0.035	0.126	-0.112	-0.424	0.339
hsa-miR-1207-5p	0.387	0.364	0.124	0.396	0.170	0.466	-0.033	-0.153	0.207	-0.170	-0.065	-0.094
hsa-miR-1225-5p	0.116	0.191	-0.159	0.401	0.138	0.520	-0.072	-0.039	-0.106	-0.453	-0.247	-0.149
hsa-miR-1246	-0.254	-0.212	-0.172	0.399	0.524	-0.118	-0.353	-0.378	-0.180	-0.583	-0.373	-0.193
hsa-miR-1249-3p	0.479	0.503	0.044	0.708	0.745	0.128	-0.615	-0.416	-0.472	-0.475	-0.415	-0.052
hsa-miR-125a-5p	0.142	0.127	-0.018	0.148	0.383	-0.310	0.021	0.162	-0.186	0.083	0.211	-0.191
hsa-miR-125b-5p	-0.504	-0.488	0.001	-0.645	-0.706	-0.110	0.083	0.354	-0.584	0.145	0.465	-0.398
hsa-miR-126-3p	-0.080	-0.050	-0.105	0.270	0.485	-0.230	0.398	0.355	0.252	0.213	0.287	-0.145
hsa-miR-1260a	-0.489	-0.470	-0.010	-0.660	-0.784	-0.021	0.013	0.171	-0.413	0.350	0.367	0.003
hsa-miR-1260b	-0.481	-0.475	0.021	-0.640	-0.800	0.043	0.065	0.235	-0.400	0.373	0.302	0.091
hsa-miR-1268b	0.444	0.504	-0.103	0.408	0.423	0.071	-0.023	-0.043	0.071	-0.292	-0.299	0.044
hsa-miR-1273g-3p	0.662	0.659	0.099	0.274	0.247	0.161	0.111	0.279	-0.134	-0.128	-0.267	0.241
hsa-miR-128-3p	0.121	-0.037	0.458	0.015	-0.047	0.264	0.405	0.386	0.167	0.338	0.169	0.121
hsa-miR-130a-3p	0.069	0.124	-0.054	0.185	0.481	-0.516	0.236	0.251	0.040	0.381	0.477	-0.156
hsa-miR-130b-3p	-0.014	-0.046	0.024	-0.342	-0.139	-0.383	-0.289	-0.251	-0.141	0.257	0.244	-0.049
hsa-miR-140-3p	-0.323	-0.232	-0.146	-0.615	-0.726	-0.001	0.142	0.088	0.079	0.479	0.079	0.413
hsa-miR-140-5p	-0.016	0.091	-0.144	-0.178	-0.223	-0.022	0.334	0.153	0.383	0.251	-0.186	0.484
hsa-miR-142-3p	-0.327	-0.251	-0.128	-0.336	-0.484	0.111	0.393	0.287	0.193	0.374	0.130	0.279
hsa-miR-142-5p	-0.237	-0.138	-0.198	-0.440	-0.590	0.116	0.457	0.276	0.409	0.404	0.144	0.277
hsa-miR-146a-5p	-0.355	-0.374	0.045	-0.319	-0.561	0.363	0.325	0.361	-0.003	0.368	0.221	0.120
hsa-miR-146b-5p	-0.268	-0.274	0.110	-0.276	-0.618	0.475	0.324	0.324	0.053	0.271	-0.037	0.354
hsa-miR-148a-3p	-0.048	-0.068	0.049	0.290	0.127	0.471	0.479	0.347	0.435	0.276	0.072	0.169
hsa-miR-148b-3p	-0.283	-0.296	0.046	0.095	0.126	-0.076	0.403	0.339	0.172	0.433	0.336	0.044
hsa-miR-150-5p	0.081	0.162	0.040	-0.181	-0.358	0.238	0.246	0.251	0.154	0.199	-0.268	0.501
hsa-miR-151a-3p	-0.053	-0.026	-0.065	0.193	0.541	-0.495	0.015	0.083	-0.118	0.108	0.314	-0.301
hsa-miR-151a-5p	-0.074	-0.088	0.061	0.396	0.694	-0.376	0.086	0.168	-0.092	0.221	0.390	-0.257
hsa-miR-151b	-0.085	-0.116	0.089	0.456	0.719	-0.292	0.173	0.257	-0.062	0.181	0.330	-0.236
hsa-miR-155-5p	-0.646	-0.652	-0.019	-0.772	-0.874	-0.060	0.147	0.159	-0.107	0.540	0.570	-0.045
hsa-miR-15a-5p	0.030	0.107	-0.099	-0.149	-0.398	0.399	0.439	0.246	0.454	0.375	0.078	0.315
hsa-miR-15b-5p	0.242	0.164	0.150	0.509	0.672	-0.117	0.386	0.471	0.030	0.403	0.300	0.069
hsa-miR-16-5p	0.322	0.415	-0.103	-0.038	-0.225	0.427	0.542	0.384	0.507	0.355	0.089	0.269
hsa-miR-17-3p	-0.227	-0.056	-0.344	-0.051	0.014	-0.206	0.294	0.116	0.322	0.548	0.212	0.336
hsa-miR-17-5p	-0.063	0.026	-0.143	-0.350	-0.195	-0.301	0.458	0.397	0.258	0.348	0.243	0.069
hsa-miR-181a-5p	0.106	0.195	-0.126	0.158	0.306	-0.272	-0.373	-0.183	-0.512	0.289	0.358	-0.128
hsa-miR-181b-5p	-0.042	-0.134	0.204	-0.202	-0.385	0.270	-0.168	-0.034	-0.341	0.371	0.374	-0.014
hsa-miR-185-5p	-0.085	-0.031	-0.125	0.109	0.355	-0.357	0.489	0.417	0.235	0.295	0.246	-0.007
hsa-miR-186-5p	-0.507	-0.494	-0.007	-0.628	-0.749	0.004	0.396	0.396	-0.027	0.394	0.233	0.171
hsa-miR-18a-5p	-0.226	-0.164	-0.148	-0.327	-0.338	-0.098	0.579	0.435	0.398	0.324	0.240	0.040
hsa-miR-18b-5p	-0.242	-0.189	-0.129	-0.215	-0.327	0.127	0.578	0.422	0.422	0.285	0.216	0.033
hsa-miR-192-5p	0.024	0.105	-0.032	-0.056	-0.262	0.299	0.371	0.230	0.390	0.190	-0.293	0.519
hsa-miR-197-3p	-0.376	-0.391	0.058	-0.452	-0.355	-0.340	-0.250	-0.044	-0.530	0.260	0.313	-0.053
hsa-miR-197-5p	0.190	0.303	-0.246	0.208	0.158	0.062	-0.177	-0.117	-0.193	-0.416	-0.321	-0.030
hsa-miR-1973	-0.152	-0.006	-0.411	0.485	0.712	-0.308	-0.345	-0.388	-0.021	-0.641	-0.307	-0.318
hsa-miR-199a-3p	-0.018	0.006	-0.047	0.307	0.552	-0.274	0.416	0.343	0.305	0.201	0.172	-0.032
hsa-miR-199a-5p	-0.255	-0.183	-0.189	-0.173	0.083	-0.472	0.487	0.502	-0.002	0.235	0.330	-0.169
hsa-miR-19a-3p	-0.080	0.033	-0.212	-0.275	-0.386	0.112	0.509	0.346	0.411	0.303	0.110	0.194
hsa-miR-19b-3p	-0.019	0.100	-0.203	-0.291	-0.406	0.121	0.463	0.328	0.364	0.266	0.051	0.214
hsa-miR-200c-3p	0.276	0.183	0.308	0.095	0.233	-0.107	-0.218	-0.043	-0.438	0.483	0.464	0.024
hsa-miR-20a-3p	-0.296	-0.243	-0.072	-0.318	-0.374	-0.037	0.287	0.169	0.220	0.348	0.142	0.216
hsa-miR-20a-5p	-0.084	0.006	-0.126	-0.325	-0.397	0.096	0.489	0.373	0.345	0.419	0.169	0.240
hsa-miR-20b-5p	-0.282	-0.240	-0.038	-0.478	-0.646	0.152	0.514	0.467	0.164	0.391	0.245	0.126
hsa-miR-21-5p	0.043	0.079	0.023	-0.049	-0.128	0.057	0.207	0.022	0.330	0.364	-0.090	0.484
hsa-miR-22-3p	-0.037	0.041	-0.178	0.154	0.394	-0.291	0.046	0.019	0.139	0.179	0.291	-0.147
hsa-miR-221-3p	-0.265	-0.298	0.023	0.016	0.312	-0.498	0.139	0.354	-0.515	0.304	0.553	-0.330
hsa-miR-222-3p	-0.434	-0.369	-0.128	-0.689	-0.793	-0.058	0.118	0.132	-0.131	0.452	0.310	0.141
hsa-miR-223-3p	-0.281	-0.164	-0.271	-0.496	-0.448	-0.246	0.627	0.524	0.238	0.315	0.227	0.064
hsa-miR-223-5p	0.093	-0.127	0.470	-0.154	-0.336	0.318	0.335	0.308	-0.001	0.298	0.247	0.043

hsa-miR-23a-3p	-0.166	-0.262	0.243	0.006	0.118	-0.183	0.175	0.229	-0.076	0.418	0.360	0.018
hsa-miR-23b-3p	-0.387	-0.387	-0.046	-0.148	0.116	-0.501	0.007	0.175	-0.262	0.236	0.390	-0.210
hsa-miR-24-3p	0.199	0.272	-0.094	0.190	0.406	-0.306	0.408	0.306	0.314	0.371	0.222	0.098
hsa-miR-25-3p	0.013	0.096	-0.046	-0.534	-0.556	0.005	0.398	0.367	0.126	0.360	0.148	0.202
hsa-miR-26a-5p	-0.358	-0.263	-0.168	-0.571	-0.538	-0.257	0.245	0.301	-0.095	0.420	0.354	0.043
hsa-miR-26b-5p	-0.102	-0.083	0.099	-0.293	-0.646	0.526	0.594	0.420	0.523	0.513	0.156	0.357
hsa-miR-27a-3p	-0.062	0.090	-0.304	0.146	0.273	-0.263	0.334	0.141	0.405	0.464	0.207	0.231
hsa-miR-27b-3p	-0.389	-0.280	-0.266	-0.188	0.015	-0.445	0.257	0.250	0.107	0.284	0.324	-0.096
hsa-miR-28-5p	-0.004	0.097	-0.064	0.107	0.080	-0.029	0.326	0.153	0.442	0.446	-0.021	0.492
hsa-miR-2861	-0.351	-0.287	-0.118	-0.206	-0.411	0.212	0.099	0.040	0.081	-0.148	0.016	-0.158
hsa-miR-29a-3p	0.009	0.172	-0.252	-0.117	-0.335	0.313	0.508	0.322	0.581	0.289	-0.259	0.577
hsa-miR-29b-3p	-0.134	-0.029	-0.159	-0.299	-0.551	0.322	0.476	0.228	0.600	0.307	-0.175	0.496
hsa-miR-29c-3p	-0.214	-0.072	-0.243	-0.350	-0.588	0.281	0.531	0.306	0.590	0.285	-0.153	0.444
hsa-miR-301a-3p	-0.090	0.013	-0.206	-0.017	0.016	-0.093	0.331	0.201	0.252	0.370	0.266	0.098
hsa-miR-30b-5p	-0.385	-0.307	-0.134	-0.538	-0.661	0.024	0.397	0.296	0.146	0.401	0.215	0.171
hsa-miR-30c-5p	-0.525	-0.486	-0.079	-0.563	-0.638	-0.072	0.322	0.337	-0.168	0.473	0.406	0.061
hsa-miR-30d-5p	-0.034	-0.058	0.077	0.405	0.682	-0.294	-0.316	-0.153	-0.440	0.271	0.406	-0.167
hsa-miR-30e-3p	-0.242	-0.148	-0.138	-0.142	-0.189	-0.016	0.188	0.044	0.202	0.468	0.043	0.439
hsa-miR-30e-5p	-0.238	-0.122	-0.212	-0.525	-0.651	0.063	0.504	0.355	0.318	0.356	0.125	0.232
hsa-miR-3162-5p	0.458	0.511	-0.075	0.104	0.098	0.038	-0.047	-0.051	0.035	-0.158	-0.348	0.241
hsa-miR-3195	0.515	0.535	-0.007	0.396	0.429	0.062	-0.214	-0.116	-0.135	-0.522	-0.618	0.185
hsa-miR-3196	-0.231	-0.203	0.011	-0.515	-0.654	0.059	-0.149	-0.114	-0.184	0.057	0.021	0.096
hsa-miR-3198	0.613	0.681	-0.073	0.453	0.508	-0.029	0.056	0.096	-0.015	-0.511	-0.713	0.251
hsa-miR-320a	0.307	0.253	0.173	0.155	0.537	-0.617	-0.326	-0.072	-0.577	0.148	0.398	-0.318
hsa-miR-320b	0.384	0.324	0.203	0.135	0.486	-0.560	-0.275	-0.002	-0.523	0.079	0.385	-0.377
hsa-miR-320c	0.423	0.355	0.223	0.191	0.496	-0.460	-0.223	0.038	-0.472	-0.006	0.253	-0.306
hsa-miR-320d	0.451	0.355	0.290	0.180	0.521	-0.514	-0.264	0.005	-0.494	-0.028	0.265	-0.347
hsa-miR-320e	0.424	0.321	0.307	0.138	0.467	-0.490	-0.267	0.013	-0.527	0.040	0.290	-0.299
hsa-miR-324-3p	-0.474	-0.464	-0.043	-0.682	-0.788	-0.091	0.284	0.491	-0.455	0.461	0.573	-0.159
hsa-miR-324-5p	0.135	0.113	-0.030	0.353	0.643	-0.357	-0.033	0.075	-0.184	0.325	0.417	-0.170
hsa-miR-326	-0.369	-0.343	-0.141	0.105	0.328	-0.367	0.114	0.205	-0.145	0.214	0.486	-0.362
hsa-miR-331-3p	-0.132	-0.214	0.124	0.402	0.672	-0.312	-0.084	0.047	-0.173	0.397	0.426	-0.070
hsa-miR-335-5p	-0.326	-0.253	-0.157	-0.243	-0.127	-0.235	0.592	0.584	0.156	0.412	0.430	-0.058
hsa-miR-338-3p	-0.486	-0.378	-0.339	-0.334	-0.355	-0.021	0.283	0.172	0.164	0.364	0.176	0.167
hsa-miR-340-3p	-0.189	-0.199	0.075	-0.206	-0.255	0.025	0.420	0.347	0.119	0.469	0.321	0.131
hsa-miR-340-5p	-0.105	-0.021	-0.206	-0.406	-0.564	0.187	0.579	0.360	0.513	0.382	0.241	0.101
hsa-miR-342-3p	0.284	0.401	0.046	-0.167	-0.309	0.168	0.064	0.127	-0.013	0.426	0.011	0.448
hsa-miR-342-5p	0.619	0.490	0.543	0.129	0.018	0.233	-0.006	0.035	0.066	0.112	-0.325	0.497
hsa-miR-361-3p	0.200	0.406	-0.211	-0.418	-0.554	0.124	0.094	0.071	0.152	0.275	-0.026	0.306
hsa-miR-361-5p	0.392	0.491	0.013	-0.109	0.195	-0.512	-0.467	-0.255	-0.525	0.321	0.291	-0.007
hsa-miR-362-5p	0.290	0.422	-0.228	-0.313	-0.177	-0.331	-0.180	-0.233	-0.093	0.220	-0.032	0.267
hsa-miR-363-3p	-0.405	-0.310	-0.198	-0.317	-0.528	0.328	0.072	0.175	-0.159	0.115	0.106	-0.027
hsa-miR-3651	0.718	0.683	0.239	0.125	0.025	0.206	-0.063	0.104	-0.315	0.217	-0.087	0.295
hsa-miR-3653-3p	0.607	0.579	0.312	0.123	0.214	-0.151	-0.496	-0.366	-0.366	0.113	-0.331	0.493
hsa-miR-3656	0.362	0.421	-0.080	0.458	0.454	0.190	-0.166	-0.117	-0.143	-0.370	-0.223	-0.114
hsa-miR-3665	0.178	0.204	0.000	0.005	-0.250	0.427	0.077	0.050	0.100	0.132	0.135	0.016
hsa-miR-374a-5p	-0.162	-0.009	-0.306	-0.446	-0.518	-0.022	0.704	0.545	0.475	0.427	0.223	0.186
hsa-miR-374b-5p	-0.346	-0.236	-0.260	-0.678	-0.549	-0.318	0.622	0.582	0.242	0.329	0.302	-0.009
hsa-miR-376a-3p	-0.023	0.074	-0.093	0.296	0.272	0.066	0.063	-0.006	0.253	0.004	0.104	-0.171
hsa-miR-376c-3p	0.106	0.188	-0.040	0.335	0.328	0.049	0.056	-0.004	0.256	-0.008	0.008	-0.071
hsa-miR-378a-3p	0.634	0.564	0.303	-0.013	-0.093	0.190	-0.379	-0.364	-0.291	-0.060	-0.255	0.203
hsa-miR-378d	0.234	0.116	0.377	-0.124	-0.333	0.379	-0.175	-0.177	-0.204	0.030	-0.180	0.219
hsa-miR-378i	0.594	0.493	0.384	-0.078	-0.192	0.227	-0.342	-0.314	-0.310	-0.047	-0.221	0.181
hsa-miR-3960	-0.383	-0.414	0.073	-0.338	-0.395	-0.076	0.068	0.100	-0.246	0.189	0.248	-0.045
hsa-miR-423-3p	0.135	0.297	-0.331	0.394	0.684	-0.421	-0.343	-0.211	-0.347	0.112	0.416	-0.313
hsa-miR-423-5p	0.626	0.501	0.388	0.229	0.488	-0.326	-0.305	0.016	-0.655	0.254	0.258	-0.065
hsa-miR-424-5p	-0.460	-0.424	-0.117	-0.307	-0.492	0.199	0.368	0.254	0.286	0.051	-0.166	0.221
hsa-miR-425-5p	-0.185	-0.176	-0.069	-0.462	-0.358	-0.209	0.422	0.431	-0.026	0.380	0.333	0.002
hsa-miR-4281	0.042	0.016	0.138	0.105	-0.198	0.496	-0.104	-0.145	0.008	-0.210	-0.039	-0.148
hsa-miR-4284	0.831	0.696	0.472	-0.016	-0.177	0.365	0.077	0.183	-0.128	0.342	0.011	0.333
hsa-miR-4286	0.366	0.252	0.323	-0.595	-0.672	-0.007	-0.029	0.068	-0.229	0.183	0.043	0.142
hsa-miR-4291	-0.171	-0.069	-0.246	-0.206	-0.032	-0.387	0.121	0.118	-0.114	0.381	0.336	-0.035
hsa-miR-4299	-0.097	0.035	-0.309	0.271	0.074	0.269	0.116	0.212	-0.188	-0.341	-0.198	-0.063
hsa-miR-4306	0.248	0.218	0.089	0.486	0.706	-0.163	0.328	0.257	0.268	0.199	0.080	0.075
hsa-miR-4323	0.268	0.320	0.088	-0.175	0.079	-0.497	-0.558	-0.291	-0.712	-0.069	-0.056	0.013
hsa-miR-4442	0.569	0.614	-0.001	0.304	0.282	0.048	-0.054	-0.062	-0.006	-0.412	-0.380	0.016
hsa-miR-4443	0.423	0.455	-0.032	0.423	0.475	0.014	-0.430	-0.500	0.166	-0.468	-0.461	0.090
hsa-miR-4459	0.225	0.300	-0.164	0.479	0.619	-0.138	-0.281	-0.271	-0.130	-0.506	-0.367	-0.083
hsa-miR-4466	-0.213	-0.110	-0.227	0.070	-0.203	0.385	0.122	0.015	0.189	-0.069	0.003	-0.057

hsa-miR-4485-3p	0.040	0.186	-0.364	0.528	0.771	-0.309	-0.386	-0.411	-0.074	-0.631	-0.325	-0.278
hsa-miR-4485-5p	0.475	0.467	0.064	0.578	0.840	-0.335	-0.534	-0.499	-0.193	-0.709	-0.337	-0.363
hsa-miR-4505	-0.052	0.126	-0.411	0.293	0.225	0.106	-0.137	-0.186	0.052	-0.430	-0.301	-0.123
hsa-miR-4507	0.266	0.391	-0.260	0.470	0.477	0.083	-0.250	-0.294	0.092	-0.463	-0.350	-0.087
hsa-miR-4516	-0.458	-0.435	-0.088	-0.439	-0.322	-0.376	0.046	-0.002	-0.057	-0.049	-0.095	0.050
hsa-miR-451a	-0.721	-0.610	-0.403	-0.254	-0.367	0.025	0.339	0.463	-0.146	0.241	0.300	-0.034
hsa-miR-4530	0.282	0.407	-0.259	0.562	0.541	0.149	-0.128	-0.177	0.086	-0.517	-0.449	-0.066
hsa-miR-454-3p	-0.236	-0.267	0.113	-0.252	-0.381	0.161	0.437	0.316	0.273	0.494	0.145	0.372
hsa-miR-4665-3p	0.416	0.446	0.042	0.652	0.524	0.416	-0.489	-0.320	-0.321	-0.140	-0.102	0.038
hsa-miR-4687-3p	0.194	0.236	-0.017	0.254	0.087	0.263	0.072	-0.007	0.126	-0.217	-0.103	-0.096
hsa-miR-4713-3p	0.634	0.684	-0.033	0.521	0.560	0.064	-0.118	-0.052	-0.021	-0.480	-0.739	0.339
hsa-miR-4739	-0.037	-0.051	0.035	-0.173	-0.428	0.328	0.333	0.289	0.139	0.386	0.389	-0.008
hsa-miR-484	-0.137	-0.198	0.097	0.244	0.489	-0.438	-0.055	0.080	-0.318	0.265	0.358	-0.129
hsa-miR-486-5p	-0.627	-0.550	-0.314	-0.139	0.105	-0.527	-0.040	0.227	-0.522	0.138	0.529	-0.418
hsa-miR-487b-3p	0.033	0.049	0.045	0.316	0.320	0.032	0.088	0.102	0.097	0.088	0.204	-0.195
hsa-miR-494-3p	-0.043	0.114	-0.390	0.382	0.597	-0.254	-0.358	-0.331	-0.209	-0.563	-0.358	-0.157
hsa-miR-5100	-0.222	-0.246	0.155	-0.597	-0.758	0.078	-0.003	0.131	-0.333	0.444	0.335	0.122
hsa-miR-532-5p	0.395	0.441	-0.025	-0.569	-0.600	-0.052	0.206	0.139	0.057	0.201	0.021	0.156
hsa-miR-5739	-0.038	-0.020	-0.017	-0.516	-0.652	0.051	-0.060	-0.130	0.103	0.067	-0.081	0.119
hsa-miR-574-5p	0.213	-0.095	0.711	-0.381	-0.152	-0.470	-0.200	-0.293	0.041	-0.500	-0.144	-0.429
hsa-miR-584-5p	-0.270	-0.406	0.206	0.087	0.193	-0.061	-0.159	0.085	-0.569	0.018	0.522	-0.613
hsa-miR-590-5p	0.005	0.121	-0.169	-0.024	-0.149	0.184	0.518	0.282	0.591	0.447	0.058	0.405
hsa-miR-6085	-0.570	-0.579	-0.013	-0.628	-0.762	0.007	0.511	0.620	-0.242	0.527	0.401	0.125
hsa-miR-6087	0.110	0.248	-0.329	0.624	0.610	0.134	-0.228	-0.287	0.035	-0.522	-0.380	-0.086
hsa-miR-6088	0.179	0.206	-0.008	0.460	0.199	0.551	-0.152	-0.231	0.123	-0.373	-0.224	-0.137
hsa-miR-6089	0.039	0.105	-0.067	0.137	-0.081	0.359	-0.080	-0.193	0.162	-0.163	-0.077	-0.089
hsa-miR-6090	-0.061	-0.025	-0.037	-0.003	-0.245	0.338	0.038	-0.026	0.043	0.038	0.067	-0.054
hsa-miR-6125	-0.530	-0.488	-0.164	-0.345	-0.570	0.257	-0.044	-0.082	-0.013	-0.213	-0.052	-0.127
hsa-miR-6126	-0.139	0.082	-0.499	-0.147	-0.292	0.142	0.030	0.094	-0.127	-0.252	-0.052	-0.184
hsa-miR-6127	0.687	0.671	0.161	0.392	0.299	0.242	-0.313	-0.196	-0.106	0.028	-0.220	0.338
hsa-miR-6131	0.702	0.697	0.105	0.534	0.519	0.181	-0.085	-0.014	-0.017	-0.348	-0.728	0.455
hsa-miR-6165	0.545	0.571	-0.009	0.160	0.218	-0.042	-0.069	-0.069	0.082	0.067	-0.278	0.354
hsa-miR-625-5p	0.233	0.088	0.447	0.364	0.401	0.084	-0.157	-0.161	0.115	0.378	0.212	0.178
hsa-miR-638	0.557	0.596	-0.006	0.640	0.646	0.150	-0.094	-0.147	0.186	-0.247	-0.238	0.024
hsa-miR-652-3p	-0.255	-0.264	-0.045	0.143	0.445	-0.439	-0.116	0.081	-0.422	0.318	0.504	-0.265
hsa-miR-660-5p	-0.001	0.076	-0.090	-0.422	-0.611	0.197	0.453	0.260	0.428	0.203	-0.091	0.308
hsa-miR-664b-3p	-0.096	-0.076	0.151	-0.222	-0.340	0.089	-0.387	-0.224	-0.396	0.170	-0.135	0.360
hsa-miR-6717-5p	0.671	0.714	0.000	0.580	0.608	0.099	-0.048	0.030	-0.020	-0.358	-0.720	0.442
hsa-miR-6740-5p	0.626	0.672	-0.024	0.574	0.608	0.080	-0.125	-0.118	0.046	-0.551	-0.784	0.284
hsa-miR-6749-5p	-0.246	-0.240	-0.009	-0.510	-0.677	0.077	0.018	-0.077	0.162	-0.013	-0.091	0.053
hsa-miR-6763-5p	0.545	0.569	-0.003	0.210	0.212	0.084	-0.036	-0.055	0.118	-0.056	-0.308	0.288
hsa-miR-6780b-5p	0.534	0.571	-0.022	0.590	0.625	0.118	-0.170	-0.128	-0.011	-0.602	-0.759	0.218
hsa-miR-6800-5p	-0.467	-0.455	-0.019	-0.337	-0.605	0.328	-0.026	-0.086	0.033	0.034	0.130	-0.095
hsa-miR-6803-5p	-0.130	0.042	-0.420	0.305	0.257	0.050	-0.065	-0.010	-0.163	-0.323	-0.109	-0.146
hsa-miR-6821-5p	-0.475	-0.424	-0.106	-0.245	-0.504	0.304	0.134	0.001	0.158	-0.064	-0.032	-0.092
hsa-miR-6826-5p	0.710	0.720	0.069	0.446	0.489	0.084	-0.439	-0.407	0.009	-0.024	-0.490	0.477
hsa-miR-6869-5p	-0.014	0.045	-0.086	0.564	0.608	0.014	-0.150	-0.322	0.271	-0.334	-0.173	-0.174
hsa-miR-6875-5p	0.663	0.663	0.090	0.591	0.566	0.219	-0.251	-0.150	-0.104	-0.487	-0.662	0.260
hsa-miR-6879-5p	0.644	0.639	0.106	0.432	0.409	0.152	-0.109	-0.052	-0.062	-0.489	-0.719	0.305
hsa-miR-7114-5p	-0.133	-0.176	0.081	-0.514	-0.669	0.088	0.119	0.204	-0.193	0.531	0.331	0.205
hsa-miR-7641	-0.139	-0.025	-0.291	0.164	0.124	0.101	-0.314	-0.218	-0.253	-0.474	-0.309	-0.096
hsa-miR-766-3p	0.117	0.145	0.038	0.167	0.472	-0.455	-0.530	-0.299	-0.509	-0.028	0.169	-0.217
hsa-miR-7704	0.218	0.233	0.020	0.247	0.082	0.383	-0.184	-0.250	0.087	-0.268	-0.208	-0.026
hsa-miR-7975	0.541	0.557	0.178	-0.138	-0.298	0.127	-0.381	-0.139	-0.602	0.426	0.139	0.311
hsa-miR-7977	-0.147	-0.164	0.096	-0.557	-0.716	0.079	-0.077	0.081	-0.368	0.467	0.302	0.164
hsa-miR-8069	0.053	0.080	0.093	-0.069	-0.443	0.534	-0.263	-0.258	-0.136	-0.002	-0.085	0.112
hsa-miR-874-3p	-0.446	-0.402	-0.059	-0.621	-0.860	0.222	-0.020	0.079	-0.422	0.154	0.373	-0.235
hsa-miR-92a-3p	-0.098	-0.107	0.125	-0.296	-0.055	-0.508	-0.201	0.050	-0.500	0.302	0.373	-0.115
hsa-miR-93-5p	0.021	0.034	-0.057	-0.260	-0.102	-0.243	0.280	0.424	-0.240	0.365	0.360	-0.047
hsa-miR-939-5p	0.474	0.479	0.024	0.114	0.006	0.225	0.030	0.070	0.000	0.113	-0.158	0.310
hsa-miR-940	-0.085	-0.050	-0.007	0.139	-0.151	0.487	-0.221	-0.040	-0.470	-0.158	-0.076	-0.036
hsa-miR-98-5p	0.000	-0.083	0.296	0.215	0.134	0.249	0.442	0.401	0.213	0.443	0.274	0.128

.
